# Alkyne-Functionalized Cyclooctyne on Si(001): Reactivity Studies and Surface Bonding from an Energy Decomposition Analysis Perspective

**DOI:** 10.3390/molecules26216653

**Published:** 2021-11-02

**Authors:** Fabian Pieck, Ralf Tonner-Zech

**Affiliations:** Wilhelm-Ostwald-Institut für Physikalische und Theoretische Chemie, Universität Leipzig, 04103 Leipzig, Germany; fabian.pieck@uni-leipzig.de

**Keywords:** density functional theory, pEDA, organic functionalization, silicon surface, nudged elastic band, ab initio molecular dynamics, chemical bonding

## Abstract

The reactivity and bonding of an ethinyl-functionalized cyclooctyne on Si(001) is studied by means of density functional theory. This system is promising for the organic functionalization of semiconductors. Singly bonded adsorption structures are obtained by [2 + 2] cycloaddition reactions of the cyclooctyne or ethinyl group with the Si(001) surface. A thermodynamic preference for adsorption with the cyclooctyne group in the on-top position is found and traced back to minimal structural deformation of the adsorbate and surface with the help of energy decomposition analysis for extended systems (pEDA). Starting from singly bonded structures, a plethora of reaction paths describing conformer changes and consecutive reactions with the surface are discussed. Strongly exothermic and exergonic reactions to doubly bonded structures are presented, while small reaction barriers highlight the high reactivity of the studied organic molecule on the Si(001) surface. Dynamic aspects of the competitive bonding of the functional groups are addressed by ab initio molecular dynamics calculations. Several trajectories for the doubly bonded structures are obtained in agreement with calculations using the nudged elastic band approach. However, our findings disagree with the experimental observations of selective adsorption by the cyclooctyne moiety, which is critically discussed.

## 1. Introduction

Silicon is already the most intensively used element for semiconductor devices [1]. Still, further development of applications in the field of solar cells, organic light emitting diodes (OLED) or molecular electronics emerge if the silicon surface is combined with organic molecules [2,3,4,5,6,7,8]. The underlying idea in this organic functionalization of semiconductor surfaces is to combine a well-established semiconductor with the chemical versatility of organic compounds [7,8,9,10].

**Figure 1:**
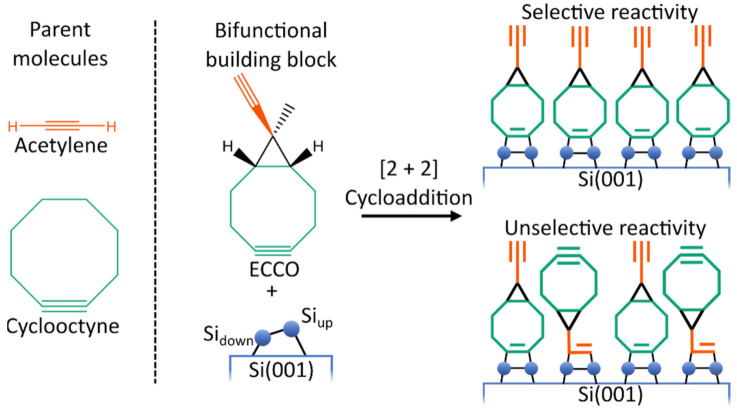
Lewis structure of the parent molecule acetylene and cyclooctyne and of 9-Ethinyl-9-Methylbicyclo[6.1.0]non-4-in (ECCO). Schematic illustration of the competitive bonding of the ethinyl (orange) and cyclooctyne (green) group, resulting in the desired selective or unwanted unselective adsorption patterns.

However, well-ordered organic layers are required to ensure proper device functionality [7,8,9,10]. This is a challenging task since the silicon surface exhibits a high reactivity toward organic molecules [11,12,13,14]. To address this challenge, bifunctional organic molecules are investigated. Here, the idea is to design an organic molecule which is suited to selectively react (Figure 1) with the silicon surface. Selectivity means that one functional group is reacting way faster with the silicon surface than the second functional group [15,16,17,18]. In a second step, the bifunctional molecule can then react with further organic molecules, introducing the desired functionality. As a mechanism for the attachment of a second organic layer, click reactions [19,20,21] are a notable approach [22,23,24,25].

From a computational point of view, the presence of two reactive groups in bifunctional molecules leads to complex reactivity patterns. Therefore, for a fundamental understanding of the reactivity of organic molecules on Si(001), it is advisable to examine monofunctional molecules first. Here, various parent molecules (i.e., molecules containing only one functional group of the bifunctional molecule) like ethylene [26,27,28,29,30,31,32,33], acetylene [27,29,30,32,34,35,36,37,38,39,40,41,42,43,44,45,46,47], cyclooctyne [30,34,48,49,50] or ethers [51,52,53,54] were studied. As it turned out, cyclooctyne is a very promising monofunctional molecule. Due to its strained triple bond [55], direct and selective adsorption was found [49,50]. Furthermore, it was shown that already adsorbed cyclooctynes can steer the adsorption of further cyclooctyne molecules [48]. Therefore, starting with cyclooctyne and adding a second functional group is a reasonable way to obtain bifunctional molecules. While the cyclooctyne group is then used for the attachment to the semiconductor surface, the second group is reserved for reactions with the molecules forming the second layer. Here, it is crucial that the second functional group reacts considerably slower with the surface than the cyclooctyne group to ensure selective adsorption (Figure 1). Following this recipe, several cyclooctyne derivates were experimentally and computationally designed and studied previously [18,22,23,41,56,57,58,59].

In the present study, the reactivity and bonding of ethinyl-cyclopropyl-cyclooctyne (ECCO, Figure 1) on Si(001) is investigated to judge its usability for the organic functionalization of semiconductor surfaces. As shown in Figure 1, ECCO is composed of cyclooctyne and acetylene functionality. Like other cyclooctyne derivates [58], the two functional groups are separated by an additional cyclopropyl element. The idea is to add some structural constraint to reduce the flexibility and reactivity of the ethinyl group in case linking to the surface via the cyclooctyne group is established. A notable characteristic of ECCO is that both functional groups comprise a C-C triple bond as the reactive functionality. The previous experimental investigation of ECCO on Si(001) showed selective adsorption by the cyclooctyne moiety [41].

In this paper, we address the competitive adsorption and reactivity of ECCO on a Si(001) surface computationally. We introduce and analyze various singly bonded adsorption structures before the reaction paths to doubly bonded structures are presented. Our results are completed by the adsorption and reaction dynamics observed in ab initio molecular dynamic (AIMD) calculations.

## 2. Methods

### 2.1. Structural Optimizations and Ab Initio Molecular Dynamic Calculations

All structures were optimized with the Vienna Ab initio Simulation Package (VASP, version 5.4.4, VASP Software GmbH, Vienna, Austria) [60,61,62,63], applying the exchange-correlation functional by Perdew, Burke and Ernzerhof (PBE) [64,65] and the DFT-D3(BJ) dispersion correction scheme with a Becke–Johnson-type damping function as developed by Grimme [66,67] et al. The electronic structure was modeled within the projector augmented wave (PAW) approach [68,69], and a plane wave basis set of 400 eV was used. A Γ-centered (2 × 2 × 1) Monkhorst-Pack grid [70] was used to sample the Brillouin zone. The wave function and forces were converged up to 10^−7^ eV and 10^−3^ eV·Å^−1^, respectively. For the selected structures, single point calculations using the range-separated hybrid functional HSE06 [71] were performed on top of the PBE optimized structure. For all minima, frequency calculations were performed. To this end, the Hessian was derived by finite differences with a displacement of 0.01 Å. Smaller imaginary modes (less than 21 cm^−1^) were attributed to numerical inaccuracy and ignored. Based on the frequency calculations, the Gibbs energies [26] at T = 300 K and p = 1 atm were calculated with statistical thermodynamics approaches. Since the presence of imaginary modes introduced small errors in the Gibbs energies (for an estimate, see Table S1), the affected energies were marked with an asterisk. The reaction paths were optimized by the nudged elastic band (NEB) method [72,73] with the climbing image (CI) method. For the NEB-CI calculations, convergence criteria of 10^−6^ eV and 10^−2^ eV·Å^−1^ were used. Prior to the NEB-CI calculations, the paths were preoptimized with a less tight criterium of 10^−1^ eV·Å^−1^ for the force convergence and without CI. In all NEB calculations, a spring force constant of 10 eV·Å^−2^ was used. AIMD calculations were performed with the velocity Verlet algorithm [74,75] (∆t = 1 fs) and the Nosé thermostat [76,77,78] (NVT ensemble, 300 K) with a mass of 1.8 (for details, see [50]). For the AIMD calculations, the wave function convergence criterium was also reduced to 10^−5^ eV. For the evaluation of the AIMD calculations, the total energy was averaged by a two-sided moving average with a range of ±510 fs. For the initial and final steps of every simulation, a corresponding one-sided moving average was used. The Si(001)-c(4 × 2) surface was modeled by a 6-layered slab (Figure S1) using a 2 × 2 supercell. The in-plane cell constant of 15.324 Å was obtained from the optimized bulk lattice of 5.418 Å. The two bottommost layers were kept fixed and saturated by hydrogen atoms with d(Si-H) = 1.480 Å [79]. In the z-direction, a lattice constant of 30.649 Å was used, ensuring a vacuum layer of at least 12 Å. These settings showed accurate results in the past [26,28,34,40,48,50,53,59].

### 2.2. Bonding Analysis

The bonding situation was analyzed by energy decomposition analysis for extended systems (pEDA) [80,81,82,83] as implemented in the program package Amsterdam Modelling Suite [84,85,86,87,88] (AMS, version 2019.305, Software for Chemistry & Materials BV, Amsterdam, Netherlands). Again, the PBE functional and the DFT-D3(BJ) dispersion correction were used. A TZ2P basis set [89] was selected, while relativistic effects were included within the zeroth-order regular approximation (ZORA) [90,91,92,93,94].

In pEDA, the bonding energy (E_Bond_) is divided into several physically well-defined terms which make it easier to understand the bonding character. The system is split into fragments that interact for bond formation. E_Bond_ can be separated (Equation (1)) into interaction energy (ΔE_int_) and preparation energy (ΔE_prep_), which is the energy difference between fragments in the structure of the final adsorbate–surface complex and the optimized structures:

(1) $$E_{\mathrm{Bond}}=\Delta E_{\mathrm{int}}+\Delta E_{\mathrm{prep}}$$

The interaction energy (ΔE_int_) can be further split into a dispersion part (ΔE_int_(disp)) and an electronic part (ΔE_int_(elec)), keeping in mind that dispersion energy also stems from electronic interactions but is evaluated in our scheme by post-Kohn Sham DFT-D3 calculations:

(2) $$\Delta E_{\mathrm{int}}=\Delta E_{\mathrm{int}}\left(\mathrm{elec}\right)+\Delta E_{\mathrm{int}}\left(\mathrm{disp}\right)$$

The electronic part (ΔE_elec_) is then separated into the electrostatic contribution (ΔE_elstat_, quasiclassical electrostatic interaction between the densities of the two fragments), Pauli repulsion (ΔE_Pauli_, antisymmetrization and normalization of the resulting product’s wave function) and orbital contribution (ΔE_orb_, charge transfer and polarization between occupied and unoccupied fragment orbitals):

(3) $$\Delta E_{\mathrm{elec}}=\Delta E_{\mathrm{elstat}}+\Delta E_{\mathrm{Pauli}}+\Delta E_{\mathrm{orb}}$$

In the pEDA, either triplet or quintet fragmentations were used, based on whether one or two triple bonds were participating in bonding to the surface. This corresponded to a shared-electron picture of the C-Si bonds.

The pEDA results were refined by the extension of the natural orbitals for chemical valence (NOCV) approach [95,96], which is currently limited to calculations at the Γ-point. The wave functions were converged to 10^−6^ a.u. For the visualization of the NOCV deformation densities, the following values for the isosurface were used for singly bonded (doubly bonded in brackets) structures: 0.001 for deformation density 1–2 (1–4), 0.0001 for density 3 (5) and 0.00005 for density 4 (6).

## 3. Results and Discussion

### 3.1. Gas Phase Structure and Adsorption Modes

Initially, the gas phase conformers of ECCO were calculated to identify the appropriate reference structure for the adsorption studies. In total, three gas phase conformers were obtained, which are shown in Figure S2. The conformers differed primarily in their structure of the cyclooctyne group. Like the parent molecule cyclooctyne [34], the cyclooctyne group of ECCO adopts conformations similar to cyclohexane. However, due to the additional constraint of the bicyclic structure, the observed conformations hardly resembled those of cyclohexane. In contrast to the parent molecule, the most stable conformer showed a boat-like conformation (C_S_ symmetry) of the cyclooctyne group. Furthermore, the cyclopropyl group was in the equatorial position, resulting in the largest observed distance (d(C-C) > 5.31 Å) between the functional groups. The least stable conformer (∆E = +47 kJ·mol^−1^) also showed a boat-like conformation, but this time, the cyclopropyl group was in the axial position. Still, C_S_ symmetry was preserved. The third conformer was best described by a chair-like conformation. This conformer was +40 kJ·mol^−1^ less stable than the most stable conformer. For the parent molecule cyclooctyne, a chair conformation was the preferred conformation [34]. This shows that the orientation of the second functional group had a significant influence on the stability of the conformers and was able to overcome the intrinsic preference of the cyclooctyne group for a chair conformation. For the adsorption studies, only the most stable conformer was used due to the significant differences in the relative energies. Therefore, all bonding energies were derived relative to this conformer. Overall, by considering only the most stable conformer, a chemical equilibrium was presumed for the gas phase.

Starting with the most stable gas phase conformer, different adsorption modes of ECCO on the Si(001) surface were obtained, which all reflected [2 + 2] cycloaddition reactions. Different cycloaddition adducts were obtained by using either the C-C triple bond of the ethinyl (E) or cyclooctyne (C) group. In addition, as for the parent molecules of cyclooctyne and acetylene, the adsorption modes differed in their positions on the surface. The on-top (OT) mode was obtained in case the C-C triple bond reacted with both atoms of one Si dimer. The bridge (BR) mode corresponded to a reaction with two Si atoms of two neighboring dimers. Additional products were obtained if a reaction with all four Si atoms of the two dimers (pedestal mode) or a reaction with an Si atom in the second layer (sublayer mode) was modeled. In the theoretical and experimental studies of the parent molecules [34,38,43,47,49], the OT and BR modes were identified as the most stable and crucial adsorption modes. Therefore, we limited the study of the competitive bonding of the functional groups of ECCO on Si(001) to these two adsorption modes.

### 3.2. Singly Bonded States

**Figure 2:**
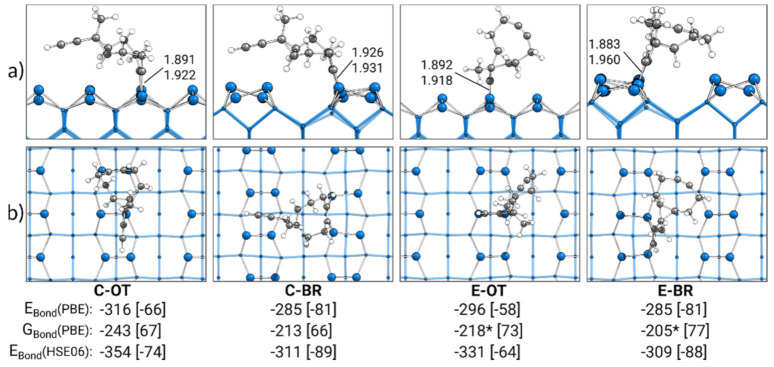
Singly bonded structures of ECCO on Si(001), with side (**a**) and top (**b**) views of the structures bonded by the cyclooctyne (C) or ethinyl (E) group in the on-top (OT) or bridge (BR) position. Electronic bonding energies (E_Bond_) are shown with their dispersion contributions in brackets. Gibbs energies (G_Bond_) are shown with their entropy contribution “−T·∆S” at 300 K and 1 atm in brackets. An asterisk marks structures with small imaginary modes (see Section 2). All energies are expressed in kJ·mol^−1^. C-Si bond lengths are expressed in Å.

In Figure 2, the most stable singly bonded structures for every adsorption mode are shown. With a bonding energy of E_Bond_ = −316 kJ·mol^−1^, the most stable structure of ECCO was found for the cyclooctyne group in the OT mode (C-OT). In case the ethinyl group was in the OT mode (E-OT), a slightly less attractive bonding energy of −296 kJ·mol^−1^ was observed. The smallest absolute value for the bonding energy of −285 kJ·mol^−1^ was observed for ECCO with either the ethinyl or cyclooctyne group in the BR mode (E-BR, C-BR). The usage of the D3(BJ) dispersion correction enabled us to identify the dispersion contribution to these bonding energies. With a range from −58 to −81 kJ·mol^−1^ (20–28%), a significant stabilization of the adsorption structures by dispersion interactions was visible, highlighting the importance of dispersion interactions in covalent surface bonding.

By calculating the Gibbs bonding energies G_Bond_, thermal and entropic corrections were also considered. In comparison with the electronic bonding energies, all Gibbs energies from −205 to −243 kJ·mol^−1^ were less attractive. Here, the main reason was the loss of translational and rotational entropy in the adsorption and hence a positive “−T·∆S” correction of 66–77 kJ·mol^−1^. As discussed in the supporting information (see Table S1), the imaginary modes for the E-OT and E-BR structures resulted in minor errors within the double-harmonic approximation in the Gibbs energy from −5 to −8 kJ·mol^−1^.

Overall, E_Bond_ and G_Bond_ showed the same trend: structures in the OT mode showed stronger bonding to the surface than structures in the BR mode. In a similar fashion, stronger bonding was observed for structures bonded with the cyclooctyne group to the surface compared with the ethinyl group.

This trend of a preference for OT over BR and C over E was also found for the parent molecules of cyclooctyne and acetylene [34]. Again, with an electronic (Gibbs) bonding energy of −308 (−238) kJ·mol^−1^, the most stable structure was observed for the cyclooctyne in the OT mode. The corresponding adsorption structure of acetylene showed a weaker bonding energy of −268 (−204) kJ·mol^−1^. The least attractive bonding energies were again observed for structures in the BR mode (cyclooctyne: −263 (−198) kJ·mol^−1^, acetylene: −249 (−187) kJ·mol^−1^). In comparison with ECCO, only a minor difference in the bonding energies of up to 36 (18) kJ·mol^−1^ was observed for the respective adsorption modes. This indicates that the two functional groups of ECCO could be understood as independent. However, a notable difference between ECCO and its parent molecules was observed for the dispersion contributions. Here, cyclooctyne and especially acetylene showed with up to −50 and −18 kJ·mol^−1^ significantly smaller dispersion interactions, respectively, in comparison with the −81 kJ·mol^−1^ of ECCO.

In conclusion, a thermodynamic preference based on the bonding energies in the competitive adsorption of ECCO for a C-OT structure was observed. Still, the difference in bonding energy to a E-OT structure was small enough that a minor fraction of E-OT adsorption structures could be expected.

### 3.3. Bonding Analysis of Singly Bonded States

To analyze the observed trends in the singly bonded structures of ECCO, pEDA was performed, and the results are shown in Table 1. This allowed us to divide the electronic interaction into meaningful attractive and repulsive contributions, while the distortion of the molecule and surface for the bond formation was quantified with the preparation energy. A discussion of the individual contributions enabled us to identify the nature of the bonding to the surface. Due to different basis sets in VASP and AMS, small differences between E_Bond_ and E_Bond_(PAW) were present. Nevertheless, the overall trend for the bonding energies between the adsorption modes was preserved.

The interaction energies (ΔE_int_) showed a different trend compared with E_Bond_ for the BR structures (C-BR: −723 kJ·mol^−1^, E-BR: −757 kJ·mol^−1^). However, the BR structures also showed larger preparation energies in comparison with the OT structures. Especially for the surface, the preparation energy was increased more than threefold in comparison with the OT structures. These larger preparation energies stemmed from a larger distortion of the Si surface, since BR adsorption distorted two Si surface dimers. Consequently, the bonding energies were less attractive for BR than for OT structures.

A smaller preparation energy was also the primary reason for a more attractive bond by C in comparison with E modes. In particular, the distortion of the molecule was smaller for adsorption by the cyclooctyne group (∆∆E_prep,**OT**_(M): −21 kJ·mol^−1^, ∆∆E_prep,**BR**_(M): −38 kJ·mol^−1^). This was caused by an already bent C-C triple bond of the cyclooctyne group and a therefore smaller additional distortion for the bond formation to the Si surface. This effect was already observed for the parent molecules [34].

While differences in the preparation energies were responsible for the preference of OT over BR and C over E, the nature of the adsorbate–surface bond was quite similar for all structures. The interaction energy was dominated by the electronic contributions, while dispersion interactions only played a minor role (≤10%). Regarding the electronic interactions, the attractive terms (ΔE_elstat_, ΔE_orb_) were slightly larger for the BR structures, while the Pauli repulsion was significantly lower. This coincided with, on average, longer Si-C bonds for the BR structures. The reduced Pauli repulsion was also the main cause for stronger electronic interactions. The covalent character was supported by the deformation densities from NOCV analysis, included in Figures S3–S6. Here, electron sharing bonds between ECCO and the Si(001) surface were observed, with a slightly stronger donation in electron density from the surface to the molecule (52% of ΔE_orb_).

In Table 1, the pEDA values of the parent molecules are also shown. In comparison with cyclooctyne, a larger interaction energy was observed for the C-OT structure. The difference in the interaction energy mainly originated from the stronger dispersion interactions for the C-OT structure, while the electronic interaction was nearly the same. In comparison with acetylene, a significantly larger dispersion interaction was also observed for the E-OT structure. However, the electronic interaction energy was also weakened. While the orbital interactions—and therefore the nature of the bond—were also similar, the electrostatic and Pauli interactions were increased. These larger electrostatic and Pauli interactions most likely stemmed from the fact that ECCO is way larger than acetylene and therefore also interacting with neighboring dimers, especially the Si_up_ atoms. This effect was also present, although less pronounced, in the comparison of cyclooctyne and acetylene [34].

Overall, the bonding analysis by the pEDA revealed that the preference of C-OT over all other adsorption modes originated mainly from the smaller preparation energy for the molecule (preference over E-OT) and the surface (preference over C-BR). The nature of the bonding to the Si surface was very similar for all adsorption modes of ECCO and also hardly differed from the bonding of the parent molecules. As a consequence, the functional groups of ECCO could be assumed to be independent in their bonding to the surface, as was also observed for another bifunctional cyclooctyne derivate [59].

### 3.4. Conformer Space

Starting from the introduced singly bonded structures, the reaction paths to the doubly bonded structures were calculated. These reaction path calculations revealed 23 conformers for the singly bonded structures. To simplify the discussion, we divided the reaction paths in the conformer changes and reactions toward doubly bonded structures. Since the barriers for conformer changes were with up to +33 kJ·mol^−1^ small, we could expect chemical equilibrium for the experimental conditions at 300 K. Therefore, the reaction barriers and energies were given relative to the corresponding conformer and not to the most stable one. However, the presence of a huge number of conformers had a significant influence on the reactivity of the adsorbates, which was not accounted for in a statistical picture. Since a reaction event is competing with the conformer changes, some time is necessary before the reaction occurs. This was especially a problem for our AIMD simulations, since we were unable to sample arbitrary long time scales. While the obtained reaction paths describing conformer changes are presented in the supporting information, the reaction paths to doubly bonded structures are introduced in the next section.

### 3.5. Doubly Bonded States

For ECCO, three types of doubly bonded structures were found, which we will discuss separately. In the following, our nomenclature for doubly bonded (DB) structures states the bonding mode of the cyclooctyne group first and then the bonding mode of the ethinyl group (e.g., DB(OT+BR) for a doubly bonded structure containing the cyclooctyne group in OT and the ethinyl group in the BR position). In this section, we will first present the reaction paths resulting in doubly bonded structures before we analyze the most stable final structures. For all reaction barriers, only the electronic energy was calculated. Since the barriers were relatively small overall, we did not expect larger changes by including Gibbs energies.

#### 3.5.1. Reaction Paths to DB(OT+BR)

**Figure 3:**
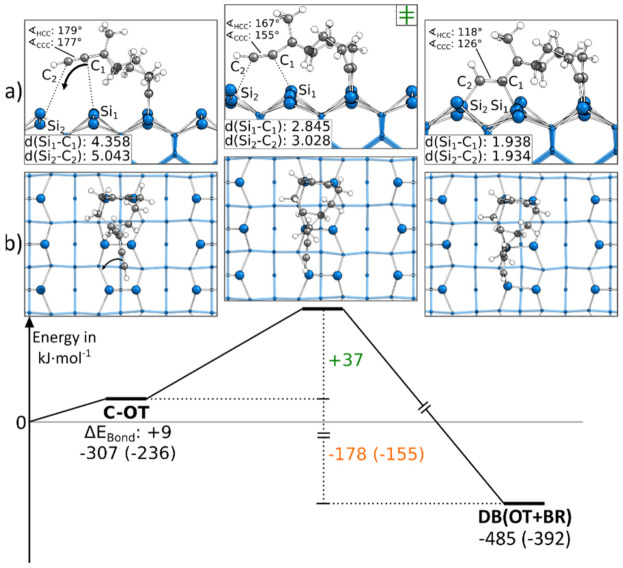
Reaction from a C-OT to a DB(OT+BR) structure, with side (**a**) and top (**b**) views of the minima and transition states. Electronic (Gibbs) bonding energies are stated for the minima. Activation energies (∆E_Act_) are shown in green, and electronic (Gibbs) reaction energies (∆E_React_) are shown in orange. All values are expressed in kJ·mol^−1^, and Si-C bond lengths are expressed in Å.

In Figure 3, the reaction to a DB(OT+BR) structure is shown, starting from a C-OT structure. For the formation of the second bond, the ethinyl group was distorted from the ideal angle of 180° while approaching the surface. Furthermore, as for all BR structures, the Si surface atoms were distorted. Overall, these deformations resulted in a minor barrier of only +37 kJ·mol^−1^. Since a second bond to the surface was formed, the reaction was with −178 kJ·mol^−1^ strongly exothermic. Figure 3 shows the reaction path in which the ethinyl group was bonded to the left Si atoms of the surface dimers. A similar reaction was present if the ethinyl group bound to the same surface dimers but the right Si atoms, as shown in Figure S12. Here, a larger barrier was observed with +52 kJ·mol^−1^.

**Figure 4:**
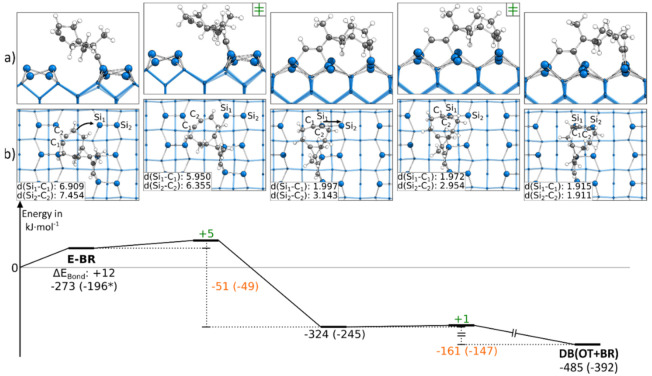
Reaction from a E-BR to a DB(OT+BR) structure, with side (**a**) and top (**b**) views of the minima and transition states. Electronic (Gibbs) bonding energies are stated for the minima. Activation energies (∆E_Act_) are shown in green, and electronic (Gibbs) reaction energies (∆E_React_) are shown in orange. All values are expressed in kJ·mol^−1^, and Si-C bond lengths are expressed in Å.

Instead of starting with the C-OT structure, the DB(OT+BR) structure could also be found starting from an E-BR structure. In Figure 4, a corresponding reaction path is shown, in which we started with the ethinyl group bonded to the left Si atoms of the surface dimers. The reaction path started by showing a rotation of the cyclooctyne group toward the neighboring surface dimer along the dimer row. This rotation showed a small barrier with +5 kJ·mol^−1^ and resulted in an intermediate state. Here, the triple bond of the cyclooctyne group was bonded to the Si_down_ atom, a common structural motif also known from acetylene, cyclooctyne and ethylene [28,40,50]. This bond resulted in an exothermic reaction step of −51 kJ·mol^−1^. The final structure of the reaction path DB(OT+BR) was then reached by pushing the cyclooctyne group in the OT position. Again, the reaction barrier with +1 kJ·mol^−1^ was negligible, and with −161 kJ·mol^−1^, a strongly exothermic step was found. In the supporting information, additional variations of this reaction path are shown, such as another reaction path (Figure S13a) for C-OT being present in case the ethinyl group was initially bonded to the right Si atoms of the surface dimers. In this case, the barrier was with +13 kJ·mol^−1^ slightly larger, and no intermediate was observed. In addition, it was possible to reach the same doubly bonded structure from a different conformer (∆E_Bond_: +10 kJ·mol^−1^) in which the cyclooctyne group was rotated. Here, the barrier dropped to around +1 kJ·mol^−1^ (Figure S13c). Furthermore, we found that a dimer flip (Figure S13b) had a negligible influence on the barrier (∆E_Act_: +4 kJ·mol^−1^).

In comparison, the barriers to reach a DB(OT+BR) structure were significantly lower in the case where ECCO was initially bonded to the surface in the E-BR instead of the C-OT mode. Furthermore, starting from the E-BR mode, the thermodynamic driving force was also lager.

#### 3.5.2. Reaction Paths to DB(BR+OT)

**Figure 5:**
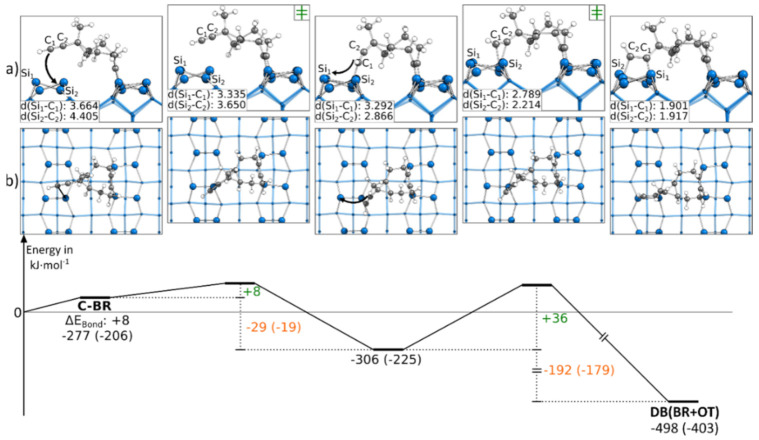
Reaction from a C-BR to a DB(BR+OT) structure, with side (**a**) and top (**b**) views of the minima and transition states. Electronic (Gibbs) bonding energies are stated for the minima. Activation energies (∆E_Act_) are shown in green, and electronic (Gibbs) reaction energies (∆E_React_) are shown in orange. All values are expressed in kJ·mol^−1^, and Si-C bond lengths are expressed in Å.

As with the first presented doubly bonded structure, the second doubly bonded structure contained one triple bond in the OT mode and one in the BR mode. In Figure 5, the reaction path to DB(BR+OT) starting from C-BR is presented. By pushing the ethinyl group close to the neighboring dimer, across the dimer row, an intermediate state is observed. Here, the terminal atom of the C-C triple bond of the ethinyl group was bonded to a Si_down_ atom. The barrier to this intermediate state was +8 kJ·mol^−1^ small, and the rate-limiting step was the consecutive reaction with a barrier of +36 kJ·mol^−1^. In this second step, the ethinyl group was pushed above the Si dimer. While the first reaction step showed with −29 kJ·mol^−1^ a minor change in the bonding energy, a strongly exothermic change of the bonding energy of −192 kJ·mol^−1^ was found for the second reaction step. The intermediate state of this reaction was only observed in case the ethinyl group was facing a Si_down_ atom. In Figure S14, the corresponding reaction, in which the ethinyl group is oriented toward the Si_up_ atom, is shown. Here, the reaction barrier with +41 kJ·mol^−1^ was slightly larger, and no intermediate was observed. Still, this reaction was also strongly exothermic with −225 kJ·mol^−1^.

For both variants of the reaction path, the initial conformer of C-BR is crucial. In all presented reaction paths, the ethinyl group was placed above the neighboring dimer row. A second set of conformers was obtained by rotating the shown C-BR structures by 180° (Figure S8b). Then, the ethinyl group was placed above the dimer row to which ECCO was bonding. For these conformers, no reactions to the doubly bonded structures were found, since it was no longer possible to bring the ethinyl group near a surface dimer.

**Figure 6:**
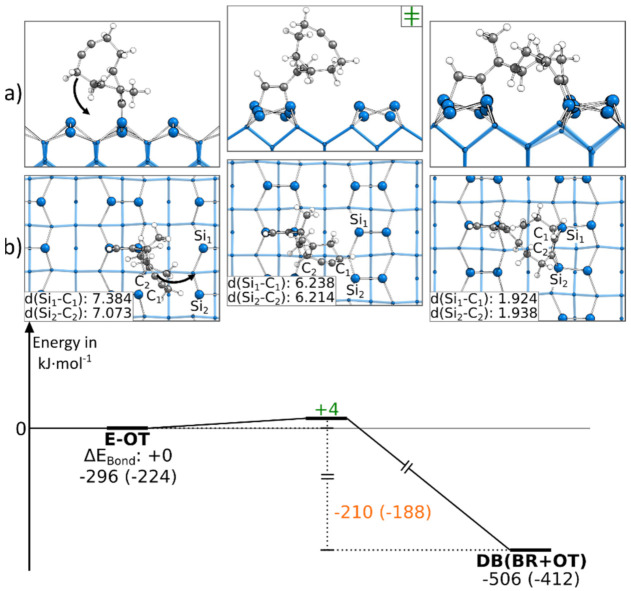
Reaction from an E-OT to a DB(BR+OT) structure, with side (**a**) and top (**b**) views of the minima and transition states. Electronic (Gibbs) bonding energies are stated for the minima. Activation energies (∆E_Act_) are shown in green, and electronic (Gibbs) reaction energies (∆E_React_) are shown in orange. All values are expressed in kJ·mol^−1^, and Si-C bond lengths are expressed in Å.

Like the first presented doubly bonded structure, the DB(BR+OT) structure could also be found starting from an E-OT structure. The corresponding reaction path is shown in Figure 6. Here, only a rotation of the cyclooctyne group was necessary to reach the final structure, leading to a small barrier of +4 kJ·mol^−1^. The bond formation also resulted in an exothermic reaction step of −210 kJ·mol^−1^. Due to the symmetry of the Si(001) surface, an analogous reaction to the neighboring dimers (Figure S15a) exhibited the same barrier and reaction energy. In Figure 6, the reaction paths, in which a Si_down_ atom is opposite the ethinyl group, is presented. A similar reaction can be observed in the case where an Si_up_ atom is opposite the ethinyl group (Figure S15b,c). Here, we were only able to find a reaction path to a DB(BR+OT) structure if, before the bond formation, the Si dimer opposite the ethinyl group was flipped (∆E_Act_: +29 kJ·mol^−1^). The consecutive rotation and bonding of the cyclooctyne group to the Si surface then had a minor barrier of +4 to +5 kJ·mol^−1^. Furthermore, we found that the symmetry- equivalent DB(BR+OT) structures could be interconverted in the case where the activation energy of +150 to +172 kJ·mol^−1^ was raised (Figure S16).

As for the reaction paths to the DB(OT+BR) structure, the DB(BR+OT) structure was most easily found in the case where ECCO was initially bonded to the E-OT structure, as shown by the significantly smaller barrier in comparison with the C-BR structure.

#### 3.5.3. Reaction Paths to DB(OT+OT)

**Figure 7:**
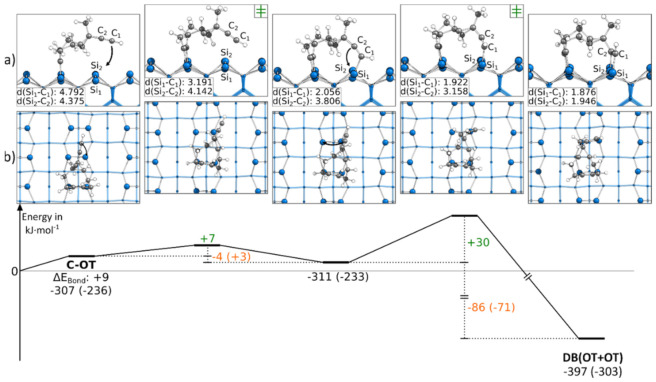
Reaction from a C-OT to a DB(OT+OT) structure, with side (**a**) and top (**b**) views of the minima and transition states. Electronic (Gibbs) bonding energies are stated for the minima. Activation energies (∆E_Act_) are shown in green, and electronic (Gibbs) reaction energies (∆E_React_) are shown in orange. All values are expressed in kJ·mol^−1^, and Si-C bond lengths are expressed in Å.

In contrast to the reactions discussed so far, Figure 7 contains a reaction path in which both triple bonds are bonding to the same adsorption mode OT. This structure was only obtained by starting from a C-OT structure. Every attempt to reach this final structure starting from an E-OT structure resulted in the formation of the DB(BR+OT) structure. Although the reaction path showed large bending of the molecule, the reaction barriers were surprisingly small with +7 and +30 kJ·mol^−1^. However, the strong distortion of the molecule resulted in smaller exothermic changes of the bonding energy by −4 and −86 kJ·mol^−1^. Furthermore, the first reaction step was the only example in which an exothermic reaction was turned into an endergonic reaction by considering the Gibbs energy. A corresponding structure in which both triple bonds were bonded to the BR mode was not found.

In conclusion, the doubly bonded structures were reachable when starting from every singly bonded adsorption mode studied. In all cases, strong exothermic and exergonic reaction steps were observed. For every singly bonded adsorption mode, at least one reaction path with a barrier of less than +36 kJ·mol^−1^ was found. In addition, alternative reaction paths with larger barriers were present. Notably, the barriers for reactions starting with an E structure were smaller than the barriers for reactions starting with a C structure. This shows that the C structures seemed to be slightly more stable and less reactive than the E structures.

### 3.6. Comparison of Doubly Bonded Structures

**Figure 8:**
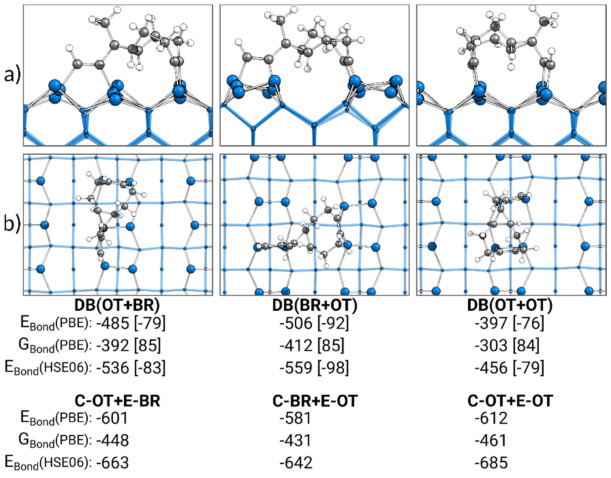
Doubly bonded structures of ECCO on Si(001), with side (**a**) and top (**b**) views of the structures bonded by the cyclooctyne (C) or ethinyl (E) group in the on-top (OT) or bridge (BR) position. Electronic bonding energies (E_Bond_) are shown with their dispersion contributions in brackets. Gibbs energies (G_Bond_) are shown with their entropy contribution “−T·∆S” at 300 K and 1 atm in brackets. In addition, the sum of the two corresponding singly bonded structures is included. All energies are expressed in kJ·mol^−1^.

In Figure 8, the most stable doubly bonded structures of each adsorption mode obtained by the discussed reaction paths are presented. Additionally, the sums of the bonding energies of the corresponding singly bonded structures are shown. Surprisingly, in comparison with the sum of the singly bonded structures, the trend for the bonding energies of the doubly bonded structures was completely reversed. Here, the DB(OT+OT) structure, at −397 kJ·mol^−1^, was the weakest doubly bonded structure and not the most stable, as the sum of C-OT and E-OT indicates. Instead, with DB(BR+OT) (−506 kJ·mol^−1^), the most stable doubly bonded structure was observed. Furthermore, DB(BR+OT) showed the smallest deviation in the electronic bonding energy from the sum of the corresponding singly bonded structures with +75 kJ·mol^−1^. This deviation rose for the DB(OT+BR) structure (+116 kJ·mol^−1^) and was the largest for the DB(OT+OT) structure (+215 kJ·mol^−1^). A smaller deviation was observed in the case where the Gibbs energies were considered, although the same trend remained. To understand this deviation for the doubly bonded structure from the sum of their singly bonded structures, pEDA was applied.

### 3.7. Bonding Analysis of Doubly Bonded States

Table 2 shows the pEDA results as differences to the sum of the corresponding singly bonded structures. The absolute values for the doubly bonded structures are shown in Table S3 in the Supporting Information. In the following discussion, we will focus on the differences between the bonding situation of the doubly bonded and the singly bonded structures. The interaction energy for the DB(BR+OT) structure showed the smallest deviation with +50 kJ·mol^−1^ in comparison with the sum of the singly bonded structures. Surprisingly, this difference stemmed mainly from a change in dispersion interaction (+45 kJ·mol^−1^), while the electronic interaction (+5 kJ·mol^−1^) was nearly identical. This negligible deviation of the electronic interaction energy was observed because the less repulsive term of the Pauli repulsion (ΔΔE_Pauli_: −48 kJ·mol^−1^) was compensated by a less attractive electrostatic and orbital interaction (ΔΔE_elstat_: +43 kJ·mol^−1^, ΔΔE_orb_: +10 kJ·mol^−1^). Furthermore, this minor difference in the electronic interactions already indicated that the bonding in the doubly bonded structure could be understood as a combination of the bonding in the singly bonded structures. The deviation in dispersion interactions originated from the point that a simple sum of the two singly bonded structures double counted the dispersion between the molecule and the surface. This effect was partly compensated by a shorter distance between the atoms of the molecule and the surface for the doubly bonded structures. In addition, the preparation energy of the DB(BR+OT) structure was +26 kJ·mol^−1^ larger than that of the sum of the individual singly bonded structures. The increased preparation energy mainly stemmed from a stronger deformation of the molecule (+21 kJ·mol^−1^). Overall, the less attractive interaction and larger preparation energy led to a smaller bonding energy in comparison with the sum of the singly bonded structures.

For the other doubly bonded structures, a similar observation could be made, although the deviations to the sum of the individual singly bonded structures were larger. For DB(OT+BR), the bonding energy was +122 kJ·mol^−1^ larger than the sum of the singly bonded structures. Again, the largest difference was observed for the dispersion interaction (+65 kJ·mol^−1^), while the preparation energy (+26 kJ·mol^−1^), and this time the electronic interaction energy (+31 kJ·mol^−1^), also contributed. For DB(OT+OT), the largest discrepancy was observed. While the sum of the contributions of the singly bonded structures indicated the most stable doubly bonded structure, the actual bonding energy was +225 kJ·mol^−1^ larger. Here, the main reason was a strong deformation of the molecule and, therefore, +136 kJ·mol^−1^ larger preparation energy. Consequently, for the electronic interaction energy (+40 kJ·mol^−1^) as well, the largest deviation was observed. However, the change in dispersion interaction was in a similar range than that for the other doubly bonded structures with +40 kJ·mol^−1^.

While the absolute values for the doubly bonded structures differed from the sum of the values of the corresponding singly bonded structures, the relative contributions to the attractive electronic interactions and to the orbital contributions were identical. This is also reflected in the NOCV deformation densities shown in Figures S17–S19, which can be understood as combinations of the deformation densities of the singly bonded structures.

In conclusion, the bonding of the doubly bonded structures can be understood as a combination of the bonding in the singly bonded structures, although some changes in the absolute pEDA values and smaller bonding energies arose due to larger structural deformations. This observation nicely agrees with previous findings for ether functionalized cyclooctyne derivates [58,59].

### 3.8. Ab Initio Molecular Dynamics

In the previous sections, we already showed that there exists a strong thermodynamic driving force for the formation of doubly bonded structures in combination with small reaction barriers. Here, we wanted to make use of AIMD simulations to complete the studies addressing the competitive bonding of the functional groups from a dynamical point of view. Therefore, 10 AIMD calculations were performed in which the adsorption and reactivity of ECCO was studied. For this, ECCO was placed 7 Å above the Si(001) surface, and an additional momentum toward the surface was added to the molecule (for details, see [50]). All obtained trajectories were included as animations in the supporting information. Before the MD simulations are presented, we want to clarify that 10 MD simulations with roughly 15 ps of simulation time are not a statistically meaningful magnitude. Therefore, the absence or presence of an observation must be discussed with caution.

An overview of the observed final structures is presented in Table 3. From the doubly bonded structures, the DB(BR+OT) structure was observed four times, while the DB(OT+BR) structure was observed once. A DB(OT+OT) structure was not observed, although the intermediate step toward this final structure was also observed once. The only singly bonded structure which was observed was the C-OT structure. However, the presence of several conformers in addition to a barrier of 37 kJ·mol^−1^ makes it unreasonable to expect the observation of a reaction event to a doubly bonded structure within 15 ps (see Table S4 for details). Since the barrier was small enough to be overcome at 300 K, a reaction event was expected for significantly longer (>400 ps) AIMD trajectories. Based on the final state distribution, a preference for doubly bonded states was indicated in these AIMD simulations. Even the observation that a singly bonded structure remained stable over a simulation time of 15 ps was rather supporting the previous findings of a slightly larger barrier than an indication for a stable singly bonded structure. In addition to the discussed final states, Table 3 also lists a “hydrogen abstraction” reaction. This describes a reaction event in which a hydrogen atom in the α position of the cyclooctyne triple bond is transferred to a Si_up_ atom. This type of reaction does not result in any [2 + 2] cycloadducts and was therefore neglected in this study.

**Figure 9:**
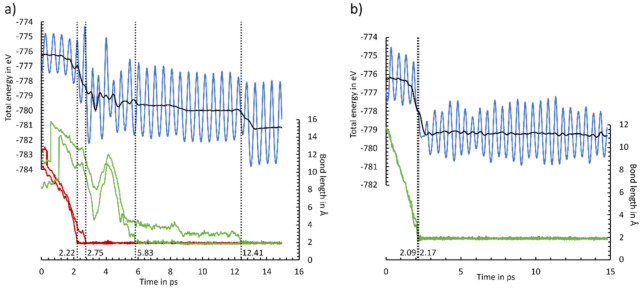
Energy (blue line) and C-Si bond length (green line: cyclooctyne group; red line: ethinyl group) changes for MD simulations 1 (**a**) and 4 (**b**). The two-sided moving average of the energy is shown as a black line. Dotted lines indicate the C-Si bond formation, stated with the time frame.

In Figure 9, two example trajectories are shown, while all other trajectories are included in the supporting information (Figure S20, Animations S1–S10). As in Figure 9, most of the AIMD simulations show direct adsorption both via the ethinyl group and the cyclooctyne group. This is indicated by a simultaneous bond formation (<0.5 ps) and an absence of any plateau in the total energy. Only two AIMD calculations of the adsorption of the cyclooctyne group (simulation 5) and the ethinyl group (simulation 6) could be understood as pseudo-direct, since a short-lived intermediate (1–3 ps) was observed. Furthermore, we observed even distributions of adsorptions via the E (6×) and C (4×) groups. Only a preference for the OT mode (9×) vs. the E mode in the adsorption was visible. Our observations are in line with the AIMD simulations of the parent molecule cyclooctyne [50]. Here, direct and pseudo-direct adsorptions with a preference for the OT mode were also found. However, a smaller difference in whether a direct or pseudo-direct adsorption was favored was present. This can be explained by the small number of AIMD simulations in both studies. A statistically meaningful number of AIMD simulations could resolve this difference. A more pronounced deviation was observed in the AIMD calculations of acetylene [40]. Here, a preference for the adsorption to a sublayer mode, which converted to the OT or BR mode, was observed. For ECCO, this adsorption mode should be less stable and therefore less likely, since the cyclooctyne group could not be properly arranged to the surface.

A natural deviation for ECCO in comparison with the parent molecules was the observation of a second reaction event. Like with the adsorption, the formation of the second bond to the final adsorption mode is also usually accomplished simultaneously (<0.5 ps). A notable exception is shown in Figure 9a. Here, an intermediate state, in which the cyclooctyne group is bonded to a Si_sublayer_ atom, is formed. This state is stable for 6.6 ps before the final BR mode is reached. This AIMD simulation supports our initial selection to focus on the BR and OT adsorption modes, while other adsorption modes are less crucial for the final product distribution.

Overall, all AIMD simulations nicely agreed with the statical picture. ECCO was found to be a highly reactive molecule. Reactions with both functional groups were likely to be expected at 300 K. A preference for adsorption via one of the functional groups was not observed. Only a preference for the OT modes, as also found in the bonding energies, was present.

### 3.9. Comparison to the Experiment

While our findings suggest that ECCO should bind to the Si surface with both functional groups whenever possible, the experimental results [41] support selective adsorption by the cyclooctyne group without consecutive reactions. In the experiment, three different combinations of temperature (T) and coverage (θ) were presented, which we want to briefly discuss.

#### 3.9.1. Low T, Low θ

Scanning tunneling microscope experiments were performed at 50 K with submonolayer (0.03 ML) coverage of cyclooctyne on Si(001) while observing C-OT. The absence of E-OT or E-BR modes was explained with a precursor state for the ethinyl group, as was found experimentally for acetylene, while C-OT reacted directly or pseudo-directly [41]. If ECCO gets trapped in the intermediate state with the ethinyl group, the conversion to C-OT has to be more likely than a conversion to E-OT or E-BR. Under this assumption, the observation of selective adsorption agrees with our data. Our bonding energies also show a thermodynamic preference for the C-OT structure while, due to the low temperature, even barriers of +37 (+30) kJ·mol^−1^ to reach DB(OT+BR) or DB(OT+OT) cannot be overcome. Therefore, the C-OT structure would stay intact, and selective adsorption would be observed.

#### 3.9.2. High T, High θ and High T, Low θ

XPS experiments were also performed for high temperatures (300 K) and nearly full monolayer or submonolayer coverage (0.13·θ_sat_). Based on the ratio of the C 1s signals, it was concluded that only singly bonded species were present. However, our data are not in agreement with this observation. For low coverages, we would expect mainly doubly bonded structures, while for high coverages, at least a distinct fraction of doubly bonded structures should be found. For high coverages, the preference of singly bonded structures could be attributed to the competition of a reaction to a doubly bonded structure and the adsorption of an additional molecule blocking the neighboring dimer atoms. Additionally, a preference for C-OT structures could be expected due to the steering of incoming adsorbates to this structure [48]. However, this cannot explain the absence of doubly bonded structures as observed in the experiment. To exclude an inaccurate preference of doubly bonded structures due to the common overbinding of the PBE functional [97], single point calculations using the HSE06 functional for the most stable singly and doubly bonded structures were performed. As shown in Figure 2 and Figure 8, even larger bonding energies were obtained with the HSE06 functional in comparison with the PBE functional. Hence, the overall thermodynamic preference for doubly over singly bonded structures was unchanged. For structures initially bonded to a surface by the cyclooctyne group, another partial explanation could be the well-known shortcoming of the PBE functional to underestimate reaction barriers [98]. Significantly larger barriers could be an explanation why for C-OT or C-BR no reactions to doubly bonded structures were observed. However, for structures bonded by the ethinyl group, this explanation is not sufficient. Even if the reaction barrier is underestimated by several factors, it would still be small enough to enable reactions at 300 K. The only possibility to exclude reactions starting from the E structures is to assume that adsorption to this structure is not taking place at all, even at 300 K. In the experiments, this was explained by the introduction of the precursor state preferring a conversion to the C-OT state. However, neither this precursor state nor the conversion was observed in our AIMD calculations. For the parent molecule acetylene, a similar discrepancy between the experiment and computations was observed and intensively discussed [40]. We assume that this still unresolved deviation between experiment and theory for acetylene adsorption is the reason why our data also deviated for ECCO.

## 4. Conclusions

We studied the competitive reactivity of the two functional groups of cyclooctyne and ethinyl in the ECCO system on Si(001). For singly bonded structures, it was shown that bonding to the on-top mode was preferred over the bridge mode. Furthermore, a thermodynamic preference for adsorption via the cyclooctyne group was found. Bonding analysis by pEDA revealed that the on-top adsorption of the cyclooctyne moiety benefitted from weaker distortions of the surface and the molecule in comparison with other adsorption modes. Still, for all adsorption modes, electron-sharing bonds with a stronger surface-to-molecule donation were found. By comparing the results of the singly bonded structures to the parent molecules, we showed that in the singly bonded structures, the functional groups behaved independently.

Starting from the singly bonded structures, several reaction paths to the doubly bonded structures were found. A higher reactivity indicated by lower barriers was found for structures initially bonded to the Si(001) surface by the ethinyl group. Furthermore, AIMD simulations supported the formation of doubly bonded structures at 300 K. With the help of pEDA, the observed doubly bonded structures were compared to the sum of their singly bonded counterparts. Here, we showed that the doubly bonded structures could be understood as a combination of two singly bonded parent structures. The observed differences in the bonding energies originated from larger distortions to form a second bond.

We thus concluded that ECCO showed singly bonded structures only at low temperatures and otherwise tended toward doubly adsorbed products. However, the double bonded structures were not consistent with the experimental observations, which showed selective adsorption of the cyclooctyne moiety in singly bonded products across different experimental conditions. We propose that the still unresolved discrepancy between experiment and computation regarding the adsorption dynamics of parent acetylene is the key to resolve this discrepancy. However, the current study does not hint toward new solutions to this old riddle.

## Figures and Tables

**Table 1:** Bonding analysis (pEDA) for the four singly bonded structure motifs of ECCO ^(a)^, pEDA values [34] and bond lengths [81] for the parent molecules cyclooctyne and acetylene in the OT mode (Cyclo. OT, Acet. OT) are included.

	C-OT		C-BR		E-OT		E-BR		Cyclo. OT		Acet. OT	
ΔE_int_	−681		−723		−677		−757		−658		−668	
ΔE _int_(disp) ^(b)^	−62	(9%)	−74	(10%)	−53	(8%)	−74	(10%)	−43	(7%)	−12	(2%)
ΔE_int_(elec) ^(b)^	−619	(91%)	−649	(90%)	−624	(92%)	−683	(90%)	−615	(93%)	−656	(98%)
ΔE_Pauli_	1500		1406		1482		1393		1468		1323	
ΔE_elstat_^(c)^	−940	(44%)	−923	(45%)	−924	(44%)	−913	(44%)	−936	(45%)	−828	(42%)
ΔE_orb_^(c)^	−1179	(56%)	−1132	(55%)	−1182	(56%)	−1163	(56%)	−1148	(55%)	−1152	(58%)
ΔE_orb_ (M→S) ^(d)^	−364	(31%)	−339	(30%)	−367	(31%)	−339	(29%)				
ΔE_orb_ (S→M) ^(d)^	−610	(52%)	−603	(53%)	−621	(53%)	−610	(52%)				
ΔE_prep_	352		429		370		464		339		389	
ΔE_prep_ (M) ^(e)^	324		329		345		367		313		364	
ΔE_prep_ (S) ^(e)^	28		100		25		97		26		25	
E_Bond_	−329		−294		−307		−293		−319		−279	
E_Bond_ (PAW)	−316		−285		−296		−285		−308		−268	
d(Si-C)	1.891		1.926		1.892		1.883		1.900		1.896	
d(Si-C)	1.922		1.931		1.918		1.960		1.916		1.913	

^(a)^ All energies are expressed in kJ·mol^−1^ and Si-C bond lengths in Å, calculated using PBE-D3/TZ2P. ^(b)^ Percentage values state the relative contributions to ∆E_int_. ^(c)^ Percentage values state the relative contributions to the sum of the attractive pEDA terms ∆E_elstat_ and ∆E_orb_. ^(d)^ Percentage values state the relative contributions to ∆E_orb_. The ∆E_orb_ term is divided into the sum of the molecule-to-surface (M→S) and surface-to-molecule (S→M) donations. ^(e)^ Contributions of preparation energy from the molecule and surface to the total preparation energy ∆E_prep_.

**Table 2:** Bonding analysis (pEDA) for the doubly bonded structure motifs ^(a)^. In this table, the difference between the sums of the two parent structures is shown. The absolute values are presented in Table S3.

	DB(OT+BR)		DB(BR+OT)		DB(OT+OT)	
ΔΔE_int_	+96		+50		+89	
ΔΔE_int_(disp) ^(b)^	+65	(−4%)	+45	(-3%)	+49	(-3%)
ΔΔE_int_(elec) ^(b)^	+31	(+4%)	+5	(+3%)	+40	(+3%)
ΔΔE_Pauli_	−77		−48		+39	
ΔΔE_elstat_^(c)^	+57	(±0%)	+43	(±0%)	+43	(−1%)
ΔΔE_orb_^(c)^	+51	(±0%)	+10	(±0%)	−42	(+1%)
ΔΔE_orb_ (M→S) ^(d)^	+31	(−1%)	+11	(±0%)	+6	(−1%)
ΔΔE_orb_ (S→M) ^(d)^	−21	(+2%)	−7	(+1%)	−57	(+1%)
ΔΔE_prep_	+26		+26		+136	
ΔΔE_prep_ (M) ^(e)^	+21		+21		+100	
ΔΔE_prep_ (S) ^(e)^	+5		+5		+36	
ΔE_Bond_	+122		+76		+225	
ΔE_Bond_ (PAW)	+116		+75		+215	

^(a)^ All energies are expressed in kJ·mol^−1^ and calculated using PBE-D3/TZ2P. ^(b)^ Percentage values state the difference in relative contributions to ∆E_int_. ^(c)^ Percentage values state the difference in relative contributions to the sum of the attractive pEDA terms ∆E_elstat_ and ∆E_orb_. ^(d)^ Percentage values state the difference in relative contributions to ∆E_orb_. The ∆E_orb_ term is divided into the sum of molecule-to-surface (M→S) and surface-to-molecule (S→M) donations. ^(e)^ Difference in contributions of preparation energy for the molecule and surface to the total preparation energy ∆E_prep_.

**Table 3:** Final configuration of ECCO in the 10 AIMD runs. The intermediate step to a DB(OT+OT) structure is labeled DB(OT+“OT”). Hydrogen abstraction indicates a structure in which hydrogen in the α position to the cyclooctyne triple bond was transferred to an Si_up_ atom.

Final Adsorption Structure	Number of Observations
C-OT	2
DB(OT+BR)	1
DB(OT+“OT”)	1
DB(BR+OT)	4
Hydrogen abstraction	2

## Data Availability

Input and output files for all calculations are available in the NOMAD repository following this DOI: https://dx.doi.org/10.17172/NOMAD/2021.09.28-2. Animations of the AIMD trajectories are available via the Zenodo repository; DOI: https://doi.org/10.5281/zenodo.5537219.

## Supplementary Materials

The following are available online. Figure S1: The Si(001)-c(4 × 2) surface model; Figure S2: Gas phase conformers of ECCO; Table S1: Uncertainty in the Gibbs energy; Figure S3–S6: NOCV deformation densities of the pEDA for single-bonded states; Table S2 and Figure S7–S11: Reaction paths for conformer changes; Figures S12–S16: Reaction paths to doubly bonded structures; Table S3: pEDA values for double-bonded structures; Figures S17–S19: NOCV deformation densities of the pEDA for double-bonded states; Table S4 and Figure S20: Ab-initio molecular dynamic simulations; Animations S1–S10: AIMD trajectories.

## Sample Availability

Samples of the compounds are not available from the authors.
