# Supplementary material for: Alkyne-Functionalized Cyclooctyne on Si(001): Reactivity Studies and Surface Bonding from an Energy Decomposition Analysis Perspective
Source: Molecules. 2021 Nov 2;26(21):6653. doi: 10.3390/molecules26216653 (PMC8586998; doi:10.3390/molecules26216653)
Supplement: Supplementary file 1 [file molecules-26-06653-s001.zip › molecules-1423139-supplementary.pdf]

# Supporting information

## Alkyne-functionalized cyclooctyne on Si(001) – reactivity studies and surface bonding from an energy decomposition analysis perspective

Fabian Pieck, Ralf Tonner-Zech*

*Wilhelm-Ostwald-Institut für Physikalische und Theoretische Chemie, Universität Leipzig, Linnéstraße 2, 04103 Leipzig, Germany, e-mail: ralf.tonner@uni-leipzig.de*

## Contents

The Si(001)-c(4x2) surface model ........ 2

Gas phase conformers of **ECCO** ........ 2

Uncertainty in the Gibbs energy ........ 2

NOCV deformation densities of the pEDA for single bonded states ........ 3

Reaction paths for conformer changes ........ 6

Reaction paths to doubly bonded structures ........ 11

pEDA values for doubly bonded structures ........ 14

NOCV deformation densities of the pEDA for double bonded states ........ 15

Ab-initio Molecular Dynamic simulations ........ 18

## The Si(001)-c(4x2) surface model

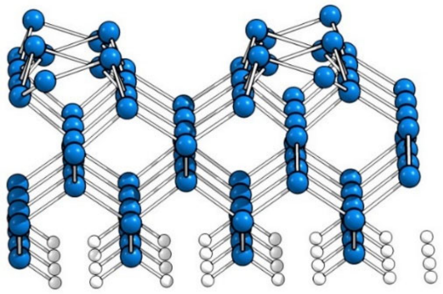

*Figure S1: Structure of the used Si(001) slab with a c(4x2) reconstruction.*

## Gas phase conformers of ECCO

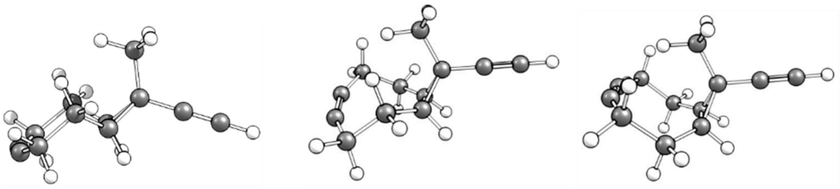

0 | +40 | +47
Used conformer

*Figure S2: Gas phase conformers of **ECCO** and their energy relative to the most stable conformer in kJ·mol$^{-1}$.*

## Uncertainty in the Gibbs energy

*Table S1: Contributions of a frequency to the enthalpy, entropy and Gibbs energy at T = 300 K and p = 1 atm. Smallest and largest observed frequency of the studied systems are shown at the bottom of the table.*

| Frequency [cm$^{-1}$] | H [kJ·mol$^{-1}$] | TS [kJ·mol$^{-1}$] | G [kJ·mol$^{-1}$] |
|---|---|---|---|
| 1 | 2 | 16 | -14 |
| 2 | 2 | 14 | -12 |
| 4 | 2 | 12 | -10 |
| 6 | 2 | 11 | -9 |
| 8 | 2 | 11 | -9 |
| 10 | 2 | 10 | -8 |
| 20 | 2 | 8 | -6 |
| 40 | 3 | 7 | -4 |
| 60 | 3 | 6 | -3 |
| 80 | 3 | 5 | -2 |
| 100 | 3 | 4 | -1 |
| 200 | 3 | 3 | 0 |
| 400 | 3 | 1 | 2 |
| 600 | 4 | 1 | 3 |
| 800 | 5 | 0 | 5 |
| 1000 | 6 | 0 | 6 |
| 2000 | 12 | 0 | 12 |
| 3000 | 18 | 0 | 18 |
| 4000 | 24 | 0 | 24 |
| 3.84 | 2 | 12 | -10 |
| 3406.63 | 20 | 0 | 20 |

For both structures **E-OT** and **E- BR** in **Figure 2** minor imaginary modes of 11 and 10 cm$^{-1}$ were observed. If we assume that the differences in electronic energies of **E-OT** and **E- BR** to the **C-OT** (ΔE = -20/-31 kJ·mol$^{-1}$) and **C-BR** (ΔE = +11/+0 kJ·mol$^{-1}$) structures are maintained in the Gibbs energies, we can estimate the introduced errors to be in the range of -5 to -8 kJ·mol$^{-1}$. This would correspond to a contribution of a real frequency in the range of 10 – 40 cm$^{-1}$ (see **Table S1**). Also, the minor imaginary modes could originate in larger real modes if overall all modes are then shifted to lower frequencies. The assumption that the trend between the electronic and Gibbs energies is approximately preserved is reasonable since all structures suffer from the exactly same loss in translational and rotational entropy.

## NOCV deformation densities of the pEDA for singly bonded states

To understand the nature of the bonding, the orbital interactions can be analyzed in greater detail by the NOCV. In **Figure S3** – **S4** the NOCVs for **C-OT**, **E-OT**, **C-BR** and **E-BR** are shown. Before the NOCVs are discussed, we want to point out that due to the missing tilting of the Si dimer for **OT** structures and the selected triplet fragmentation, two partially occupied crystal orbitals are obtained. They differ by showing either a bonding combination between the former p and p* orbitals [labeled p(Si-Si)] or an antibonding combination [labeled p*(Si-Si)]. In the NOCVs for the **C-OT** and **E-OT** structure, interactions between the π (π*) orbital of the C-C triple bond in the molecule and the formed p(Si-Si) and p*(Si-Si) orbital at the Si surface dimer are observed: The molecule donates electron density of spin β to the surface (**Figure S3/S4** II and IV), while density of spin α is donated to the molecule (**Figure S3/S4** I and III) showing the formation of electron sharing bonds. For **C-BR** and **E-BR** the same interaction is observed although now the p(Si) [p*(Si)] orbitals at the Si dimer atoms participate. Also, a clear separation of the π/ π* of the molecule is no longer present.

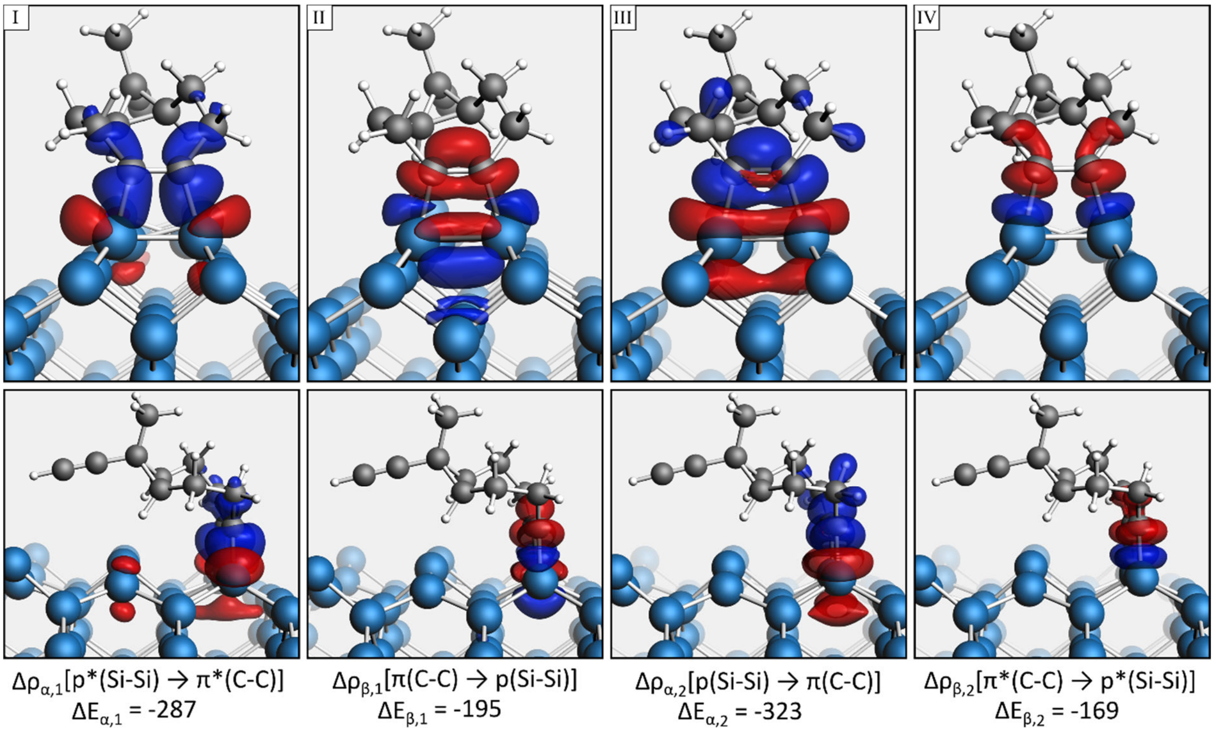


*Figure S3: NOCV deformation densities for **C-OT** based on the electron spin (α or β). Electron accumulation shown in blue and electron depletion in red. Contributions to $\Delta E_{orb}$ (ΔE) in kJ·mol$^{-1}$.*

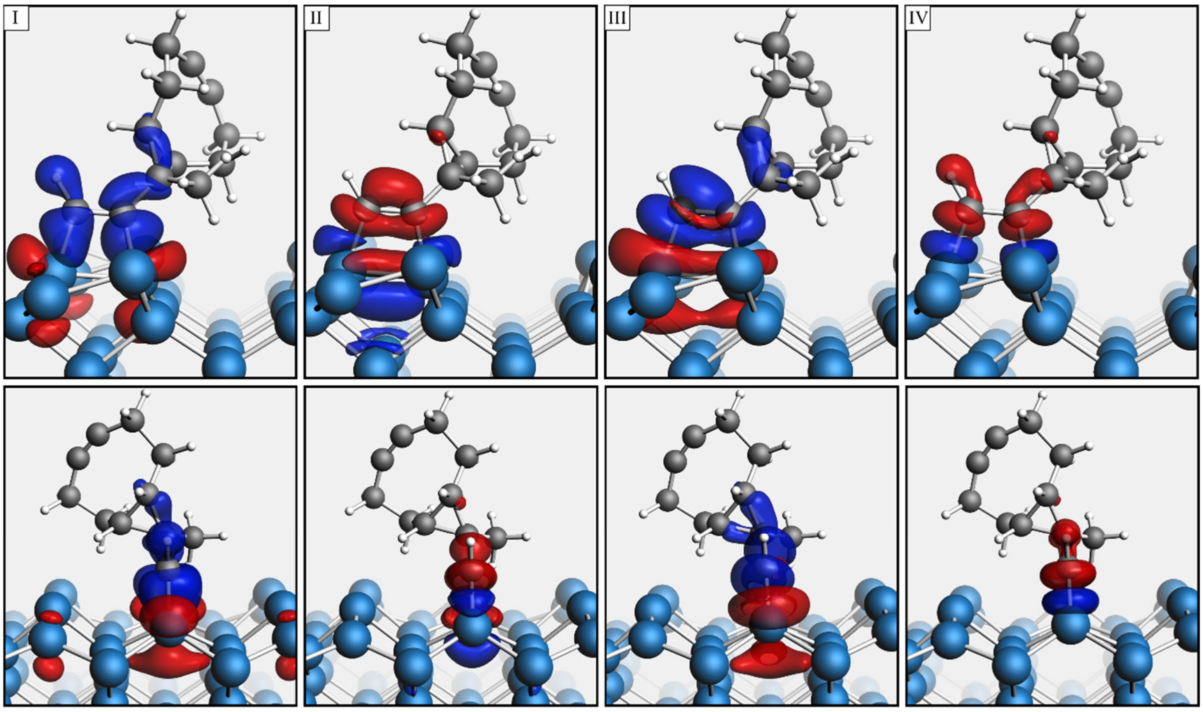


$\Delta\rho_{\alpha,1}$[π*(Si-Si) → π*(C-C)] $\nu_{\alpha,1} = \pm 0.752$ $\Delta E_{\alpha,1} = -291$

$\Delta\rho_{\beta,1}$[π(C-C) → π(Si-Si)] $\nu_{\beta,1} = \pm 0.595$ $\Delta E_{\beta,1} = -195$

$\Delta\rho_{\alpha,2}$[π(Si-Si) → π(C-C)] $\nu_{\alpha,2} = \pm 0.652$ $\Delta E_{\alpha,2} = -330$

$\Delta\rho_{\beta,2}$[π*(C-C) → π*(Si-Si)] $\nu_{\beta,2} = \pm 0.461$ $\Delta E_{\beta,2} = -172$

*Figure S4: NOCV deformation densities for **E-OT** based on the electron spin (α or β). Electron accumulation shown in blue and electron depletion in red. Eigenvalues (ν) in qe and contributions to $\Delta E_{orb}$ (ΔE) in kJ·mol$^{-1}$.*

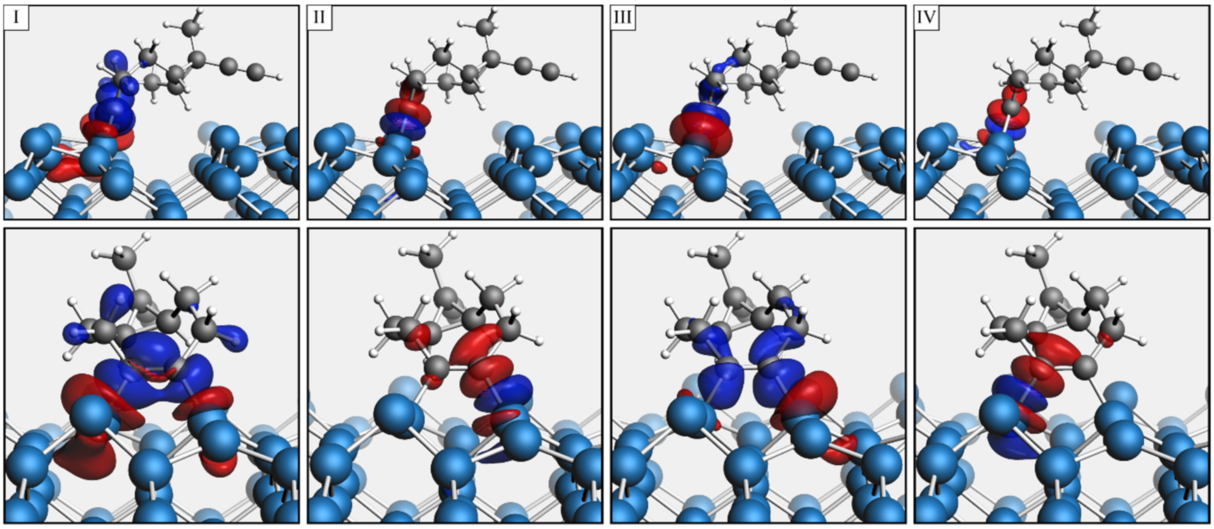


$\Delta\rho_{\alpha,1}$[p(Si)/p*(Si) → π(C-C)] $\Delta E_{\alpha,1} = -326$

$\Delta\rho_{\beta,1}$[π/π*(C-C) → p(Si)] $\Delta E_{\beta,1} = -181$

$\Delta\rho_{\alpha,2}$[p(Si) → π*(C-C)] $\Delta E_{\alpha,2} = -264$

$\Delta\rho_{\beta,2}$[π/π*(C-C) → p*(Si)] $\Delta E_{\beta,2} = -158$

*Figure S5: NOCV deformation densities for **C-Br** based on the electron spin (α or β). Electron accumulation shown in blue and electron depletion in red. Contributions to $\Delta E_{orb}$ (ΔE) in kJ·mol$^{-1}$.*

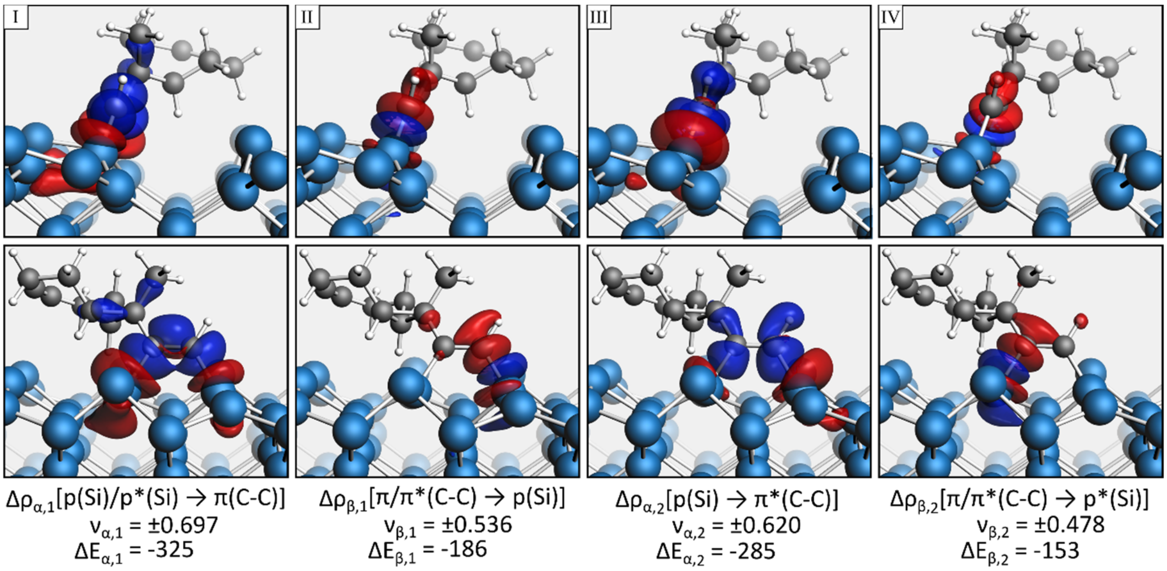


*Figure S6: NOCV deformation densities for* ***E-Br*** *based on the electron spin (α or β). Electron accumulation shown in blue and electron depletion in red. Eigenvalues (ν) in $q_e$ and contributions to $\Delta E_{orb}$ (ΔE) in kJ·mol$^{-1}$.*

## Reaction paths for conformer changes

**Table S2** contains the number of conformers per structure motif and the corresponding changes in bonding energy. The corresponding reaction paths are shown in **Figure S8**-**S11**. To simplify the discussion of the conformers a sketch showing most possibilities (**Figure S7**) is also included. The most significant distinction between the conformers is their relative position to the surface: For **C-BR** structures the ethinyl group can either be oriented above a neighboring dimer row as for the most stable conformer of this structure motif (**Figure 2**, **C-BR**) or above the dimer row on which the molecule adsorbed (**Figure S8b**). The difference between these conformers is a rotation of the adsorbate by 180°. The reaction barrier for this conversion was not calculated since it would include cleaving the strong bonds to the surface and would therefore result in a large reaction barrier. However, the presence of the second type of conformer is noteworthy since a consecutive reaction could not be found. The stability of this conformer originates in the fact that the ethinyl group cannot be brought in close proximity to an unreacted Si dimer. For **E-OT** and **E-BR** structures, different orientations on the surface are observed in the sense that the cyclooctyne group is facing either a $Si_{down}$ or $Si_{up}$ atom (for example **Figure S10a** and **S11a**). Although consecutive reactions are possible for all of these conformers, minor differences in the reaction mechanism are present. In case **ECCO** is bond to the surface by the **E** group, further conformers are observed by rotating around the C-C single bond connecting the **E** group to the cyclopropyl ring (**Figure S11b,d**). While these rotations only lead to new conformers for **E** structures, **C** structures might differ in their bending on the surface (**Figure S8, S9a**). In addition, further conformers are observed by changing the structure of the cyclooctyne, such as going from a boat-like to a chair-like structure (**Figure S8, S9b-c**). Overall, this results in plenty of different conformers as apparent in **Table S2**.

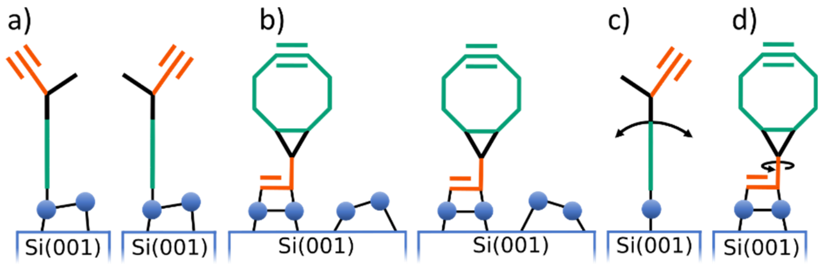


*Figure S7: Sketch of the different conformers. For **C-BR** structures different conformers are obtained based on the position of the ethinyl group towards the neighboring dimer row or above the own dimer row (a). **E-BR** and **E-OT** conformers differ in facing either a $Si_{down}$ or $Si_{up}$ atom. Further conformers are obtained by a tilting of the molecule (c) or a rotation around the C-C single bond (d). Conformer changes based on the structure of the cyclooctyne ring are not shown.*

*Table S2: Number of conformers for every structural motif. $\Delta E_{Bond}$ states the largest difference in bonding energy to the most stable conformer. $\Delta E_{Act}$ states the range of reaction barriers for a conformer change including forward and reverse reactions.*

| Structure motif | Conformers | $\Delta E_{Bond}$ | $\Delta E_{Act}$ |
|---|---|---|---|
| C-OT | 4 | 14 | 1-15 |
| C-Br | 7 | 39 | 1-33 |
| E-OT | 4 | 2 | 5-9 |
| E-Br | 8 | 33 | 1-25 |

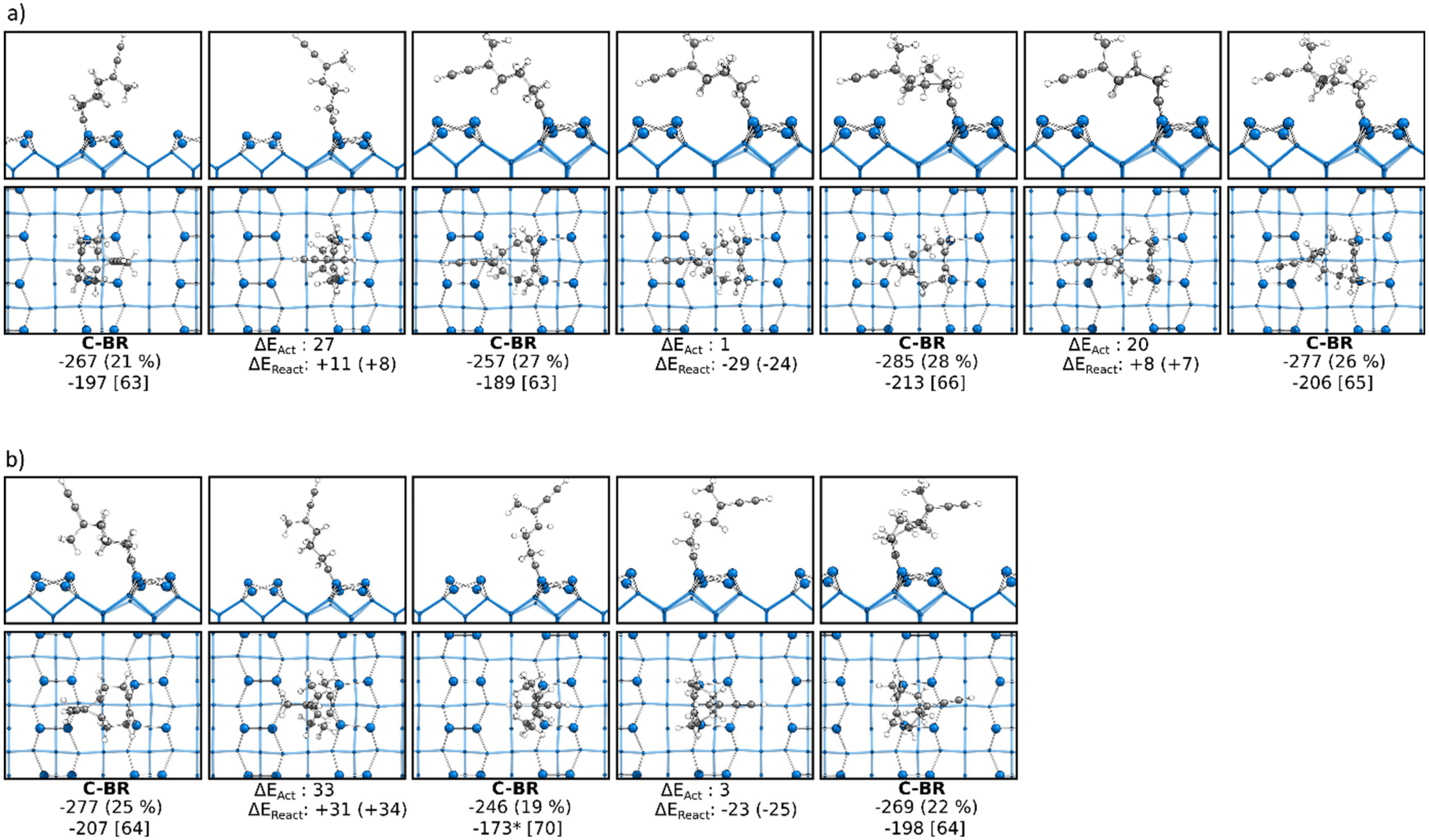


*Figure S8: Reaction paths containing conformer changes for **C-BR** structure motif. Side (top) and top (bottom) view on minima and transition states. Electronic (upper number) and Gibbs (lower number) bonding energies are stated for the minima. Activation energies ($\Delta E_{Act}$) and electronic (Gibbs) reaction energies ($\Delta E_{React}$) are stated at the transition states. All values in kJ·mol$^{-1}$.*

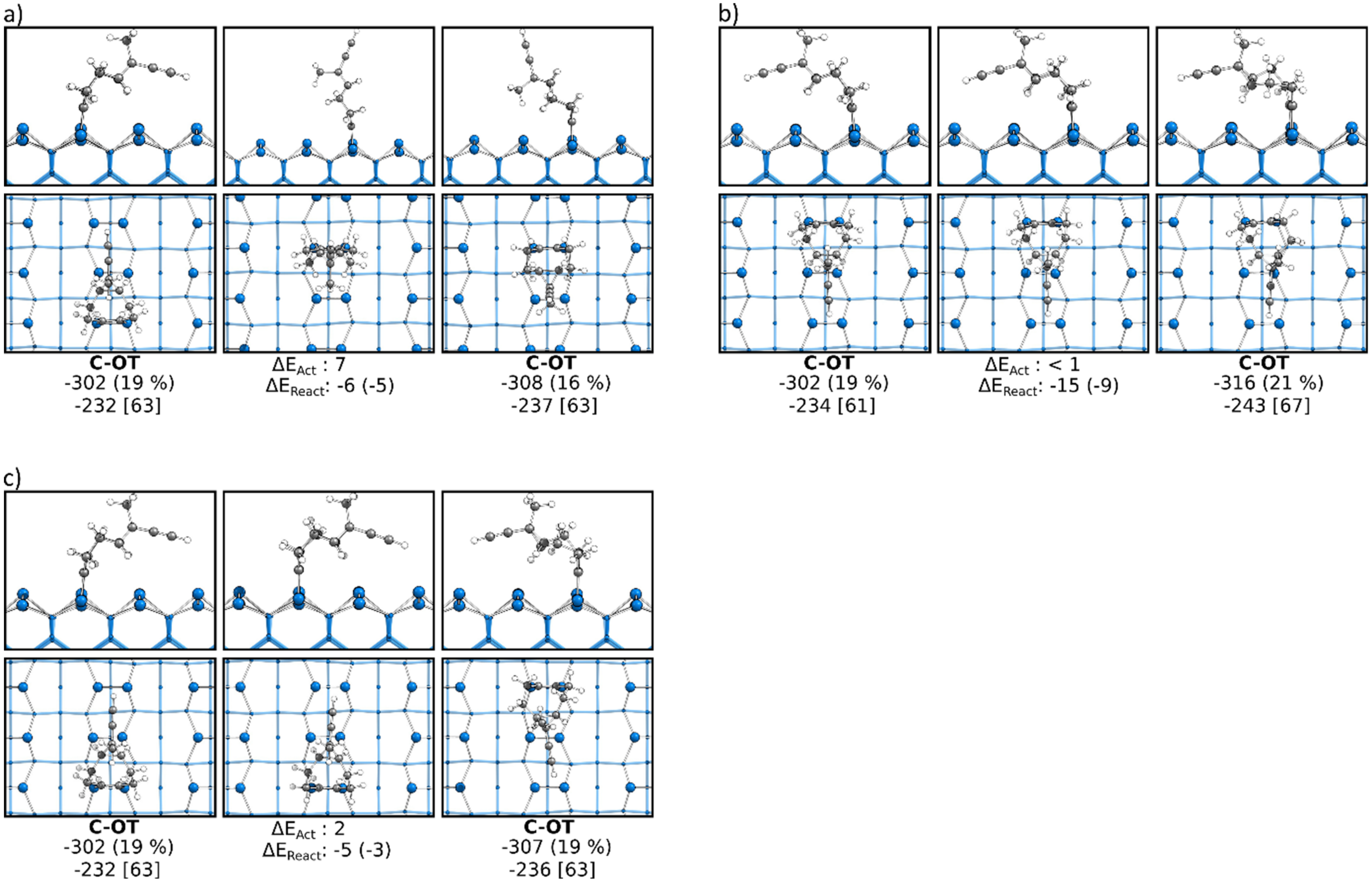


*Figure S9: Reaction paths containing conformer changes for* ***C-OT*** *structure motif. Side (top) and top (bottom) view on minima and transition states. Electronic (upper number) and Gibbs (lower number) bonding energies are stated for the minima. Activation energies ($\Delta E_{Act}$) and electronic (Gibbs) reaction energies ($\Delta E_{React}$) are stated at the transition states. All values in kJ·mol$^{-1}$.*

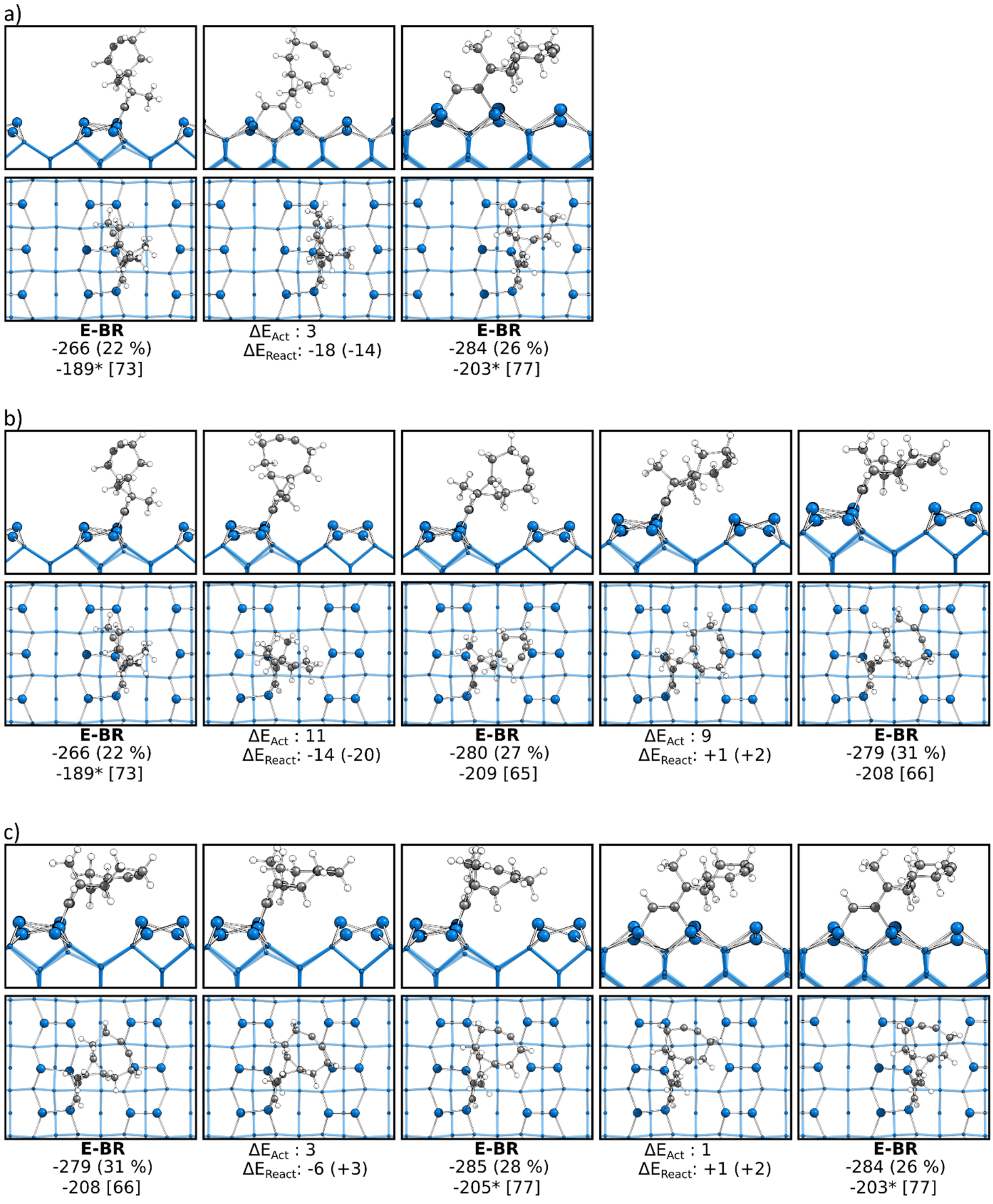


*Figure S10: Reaction paths containing conformer changes for **E-BR** structure motif. Side (top) and top (bottom) view on minima and transition states. Electronic (upper number) and Gibbs (lower number) bonding energies are stated for the minima. Activation energies ($\Delta E_{Act}$) and electronic (Gibbs) reaction energies ($\Delta E_{React}$) are stated at the transition states. All values in kJ·mol$^{-1}$.*

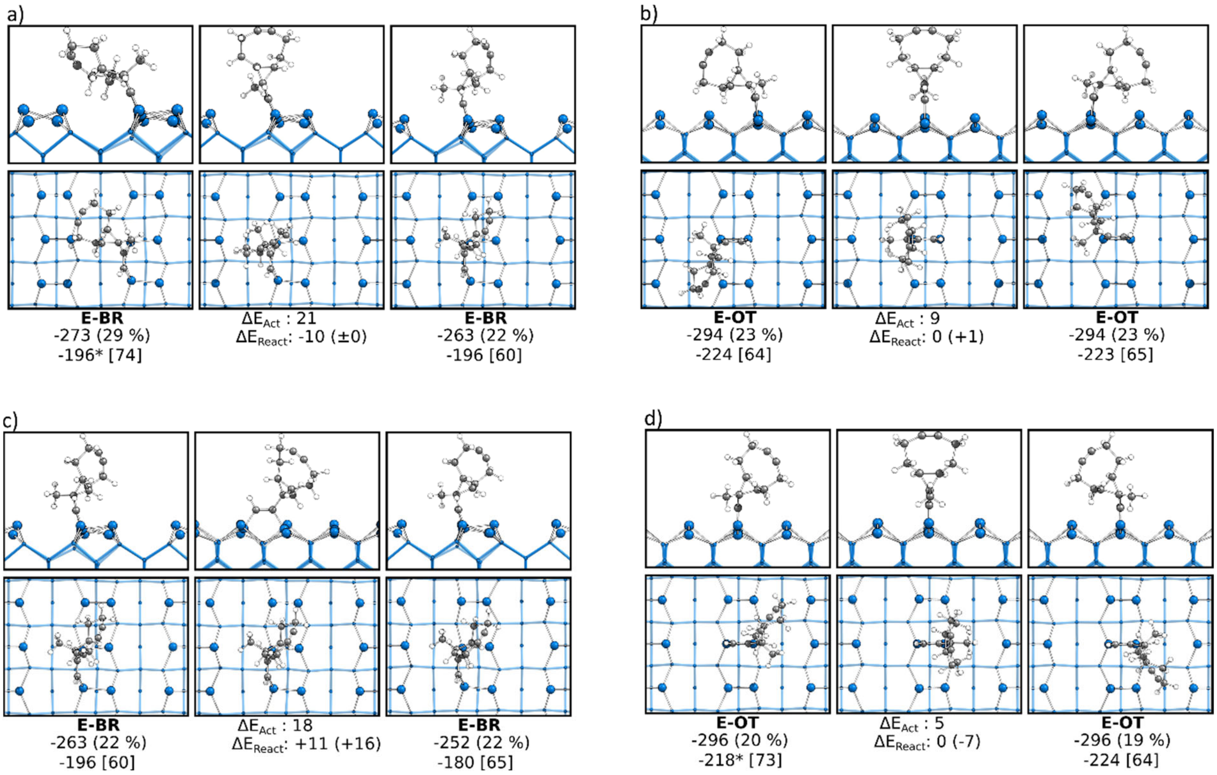


*Figure S11: Reaction paths containing conformer changes for **E-BR** and **E-OT** structure motif. Side (top) and top (bottom) view on minima and transition states. Electronic (upper number) and Gibbs (lower number) bonding energies are stated for the minima. Activation energies ($\Delta E_{Act}$) and electronic (Gibbs) reaction energies ($\Delta E_{React}$) are stated at the transition states. All values in $kJ \cdot mol^{-1}$.*

## Reaction paths to doubly bonded structures

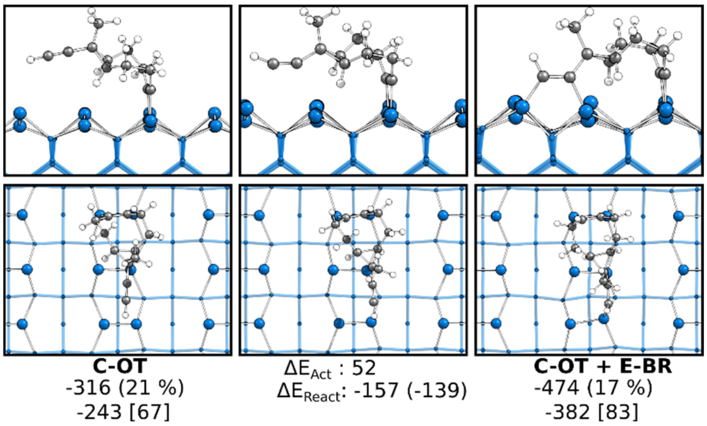


*Figure S12: Reaction from a **C-OT** to a **C-OT + E-BR** structure. Side (top) and top (bottom) view on minima and transition states. Electronic (upper number) and Gibbs (lower number) bonding energies are stated for the minima. Activation energies ($\Delta E_{Act}$) and electronic (Gibbs) reaction energies ($\Delta E_{React}$) are stated at the transition states. All values in kJ·mol$^{-1}$.*

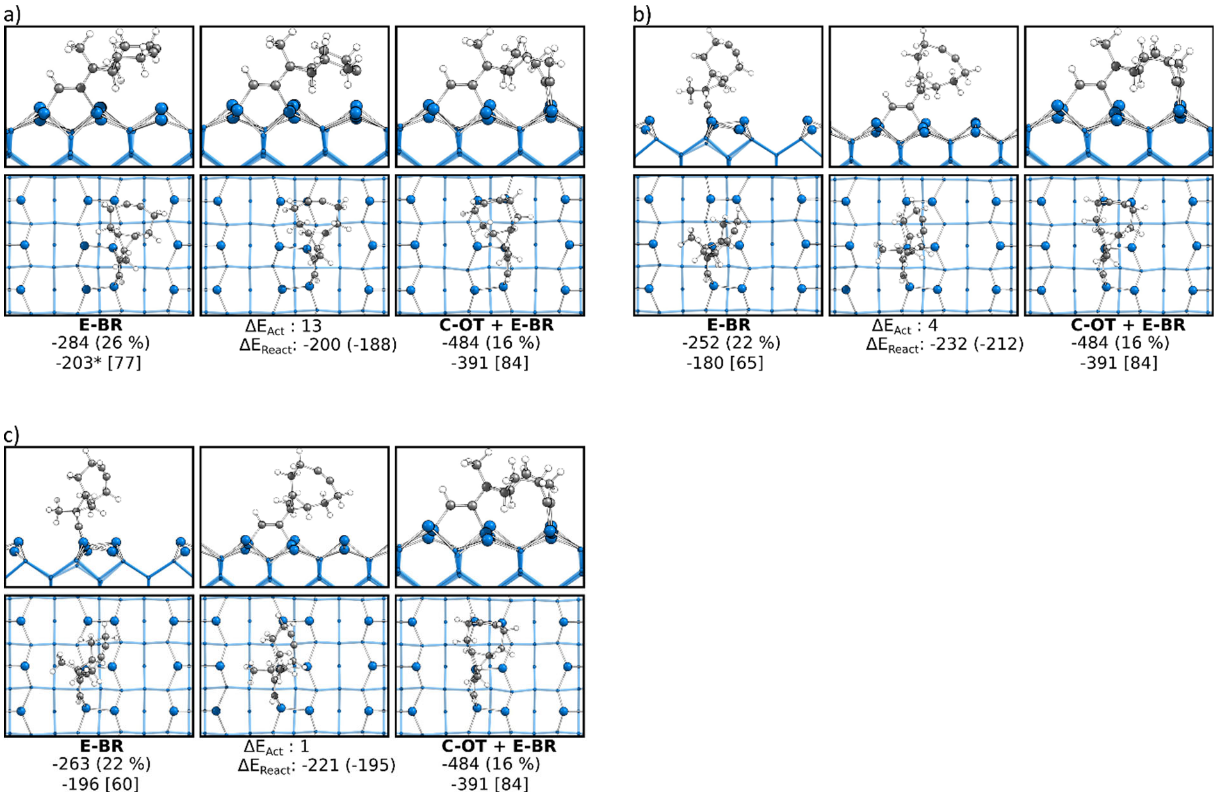


*Figure S13: Reaction from a **E-BR** to a **C-OT + E-BR** structure. Side (top) and top (bottom) view on minima and transition states. Electronic (upper number) and Gibbs (lower number) bonding energies are stated for the minima. Activation energies ($\Delta E_{Act}$) and electronic (Gibbs) reaction energies ($\Delta E_{React}$) are stated at the transition states. All values in kJ·mol$^{-1}$.*

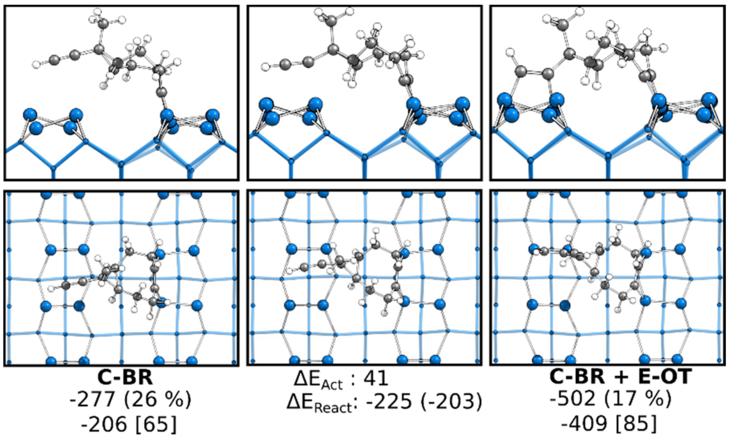


*Figure S14: Reaction from a **C-BR** to a **C-BR + E-OT** structure. Side (top) and top (bottom) view on minima and transition states. Electronic (upper number) and Gibbs (lower number) bonding energies are stated for the minima. Activation energies ($\Delta E_{Act}$) and electronic (Gibbs) reaction energies ($\Delta E_{React}$) are stated at the transition states. All values in kJ·mol$^{-1}$.*

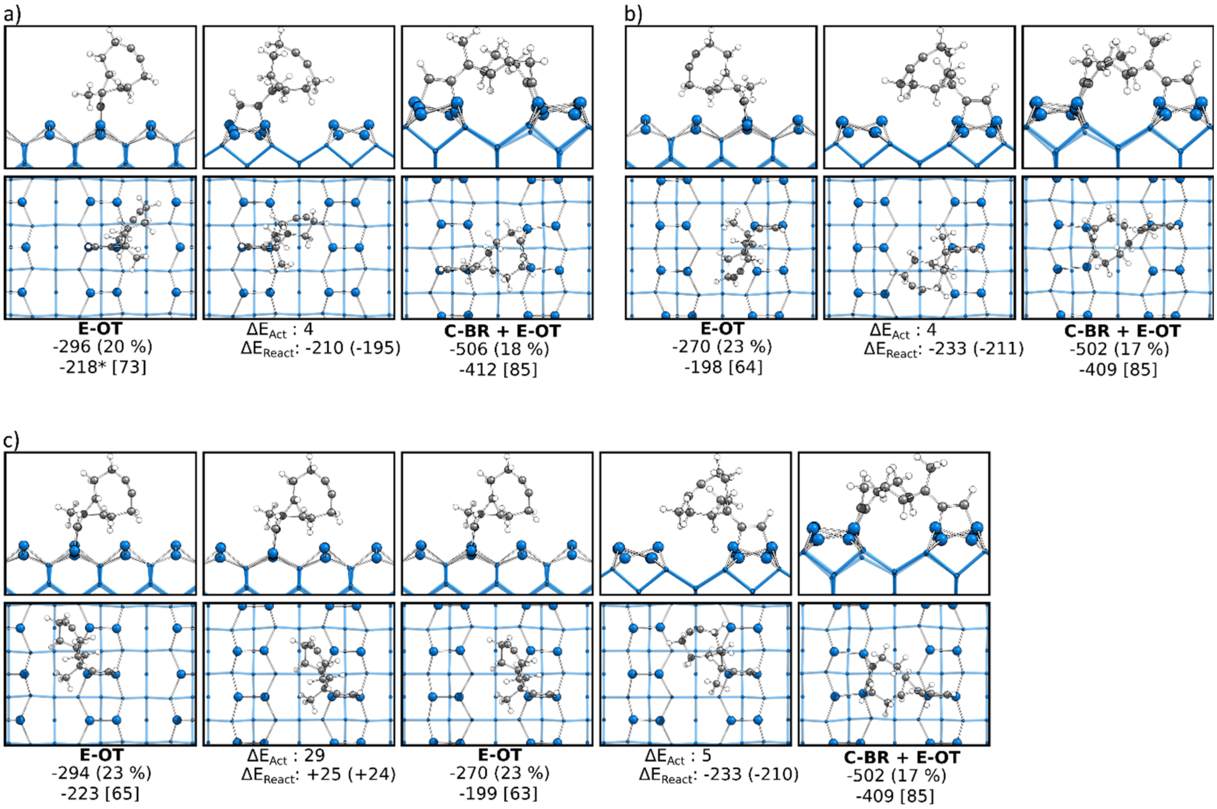


*Figure S15: Reaction from a **E-OT** to a **C-BR + E-OT** structure. Side (top) and top (bottom) view on minima and transition states. Electronic (upper number) and Gibbs (lower number) bonding energies are stated for the minima. Activation energies ($\Delta E_{Act}$) and electronic (Gibbs) reaction energies ($\Delta E_{React}$) are stated at the transition states. All values in kJ·mol$^{-1}$.*

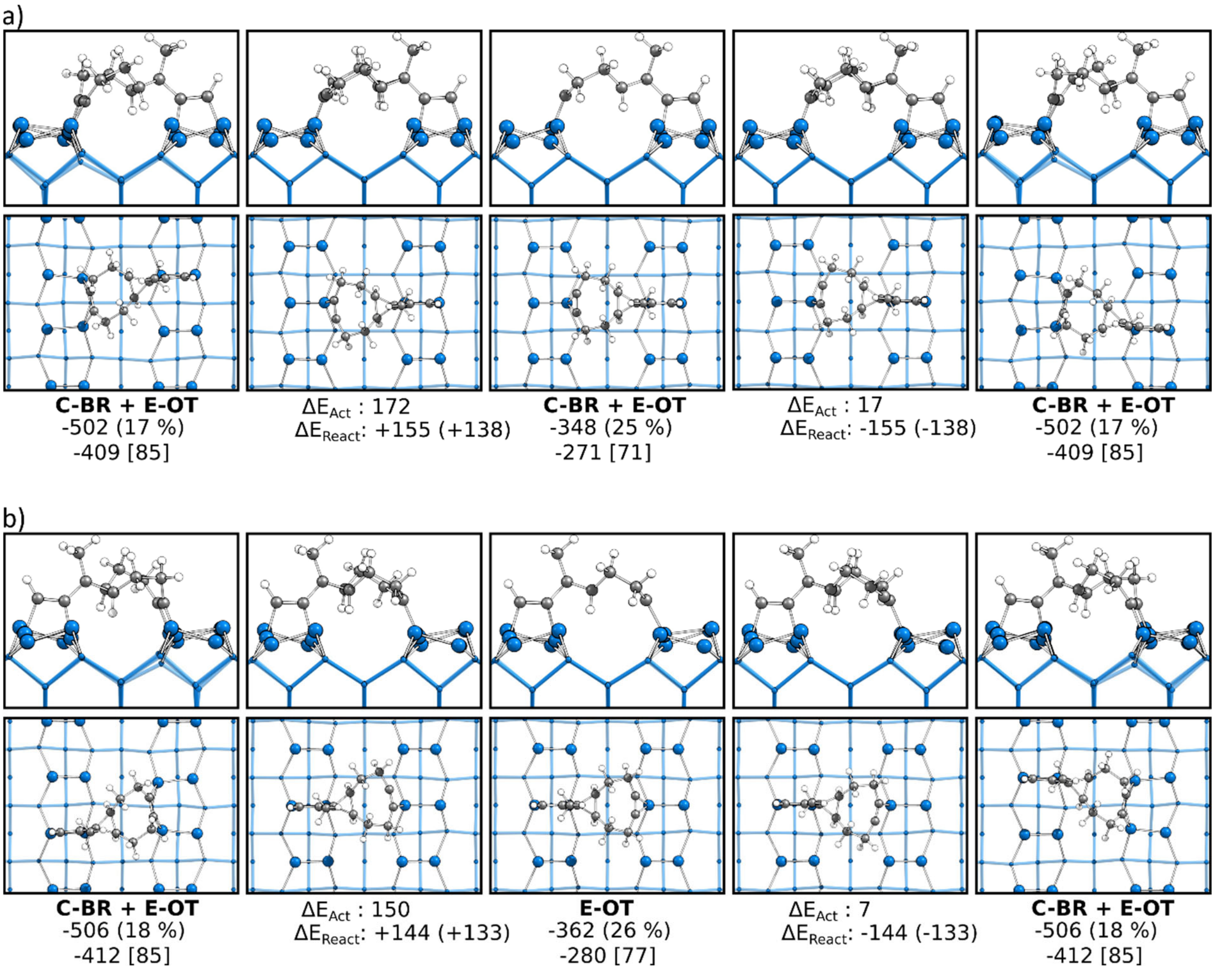


*Figure S16: Conformer changes in the* ***C-BR + E-OT*** *structure. Side (top) and top (bottom) view on minima and transition states. Electronic (upper number) and Gibbs (lower number) bonding energies are stated for the minima. Activation energies ($\Delta E_{Act}$) and electronic (Gibbs) reaction energies ($\Delta E_{React}$) are stated at the transition states. All values in kJ·mol$^{-1}$.*

## pEDA values for doubly bonded structures

*Table S3: Bonding analysis (pEDA) for the doubly bonded structure motifs. Energies in kJ·mol$^{-1}$ and bond length in Å. Percentage values state the contributions to $\Delta E_{int}$ [a], to the sum of the attractive pEDA terms $\Delta E_{elstat}$ and $\Delta E_{orb}$ [b], to $\Delta E_{orb}$ [c] and the total preparation energy $\Delta E_{prep}$ [d]. The $\Delta E_{orb}$ term is divided in molecule to surface (M→S) and surface to molecule (S →M) donations.*

| | **C-OT + E-Br** | **C-Br + E-OT** | **C-OT + E-OT** |
|---|---|---|---|
| $\Delta E_{int}$ | -1342 | -1350 | -1269 |
| $\Delta E_{int}$(disp)[a] | -71 (5%) | -82 (6%) | -66 (5%) |
| $\Delta E_{int}$(elec)[a] | -1271 (95%) | -1268 (94%) | -1203 (95%) |
| $\Delta E_{Pauli}$ | 2816 | 2840 | 3021 |
| $\Delta E_{elstat}$[b] | -1796 (44%) | -1804 (44%) | -1821 (43%) |
| $\Delta E_{orb}$[b] | -2291 (56%) | -2304 (56%) | -2403 (57%) |
| $\Delta E_{orb}$ (M→S)[c] | -673 (29%) | -696 (30%) | -725 (30%) |
| $\Delta E_{orb}$ (S→M)[c] | -1240 (54%) | -1231 (53%) | -1287 (54%) |
| $\Delta E_{prep}$ | 842 | 826 | 859 |
| $\Delta E_{prep}$ (M)[d] | 712 | 695 | 770 |
| $\Delta E_{prep}$ (S)[d] | 130 | 131 | 89 |
| $E_{bond}$ | -500 | -524 | -410 |
| d(Si-C) average | 1.925 | 1.919 | 1.913 |
| | 1.911 | 1.902 | 1.876 |
| | 1.915 | 1.910 | 1.902 |
| | 1.934 | 1.924 | 1.929 |
| | 1.938 | 1.938 | 1.946 |

## NOCV deformation densities of the pEDA for double bonded states

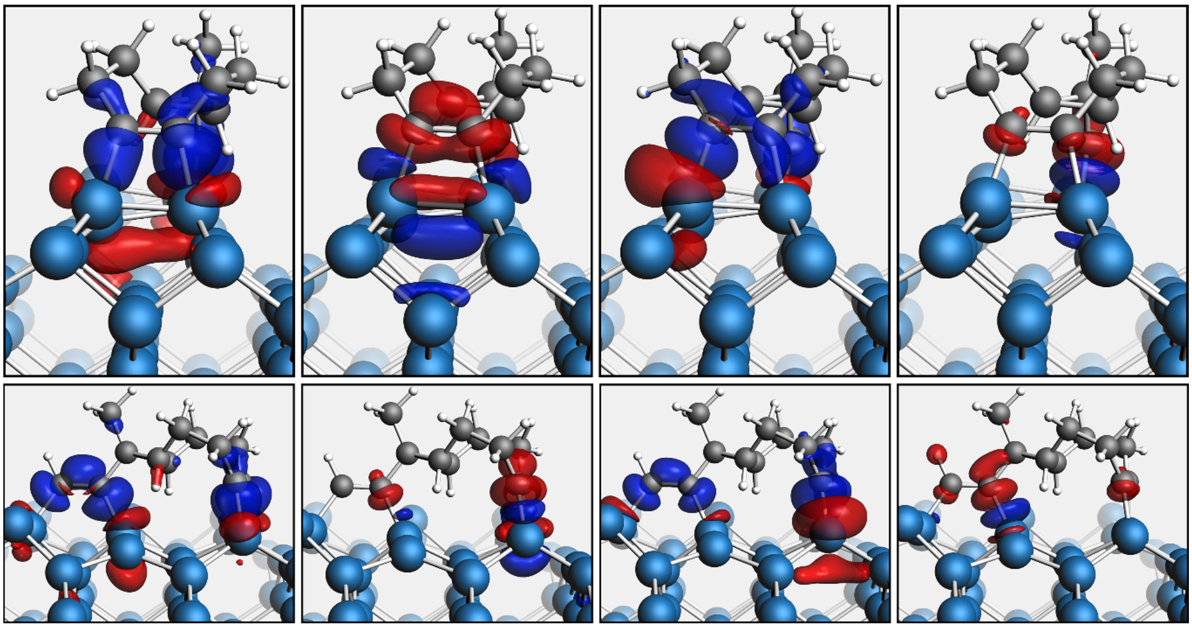

$\Delta\rho_{\alpha,1}$[**C-OT** I + **E-Br** I]
$\nu_{\alpha,1} = \pm 0.747$
$\Delta E_{\alpha,1} = -322$

$\Delta\rho_{\beta,1}$[**C-OT** II + **E-Br** IV]
$\nu_{\beta,1} = \pm 0.620$
$\Delta E_{\beta,1} = -190$

$\Delta\rho_{\alpha,2}$[**C-OT** I/III + **E-Br** I]
$\nu_{\alpha,2} = \pm 0.660$
$\Delta E_{\alpha,2} = -317$

$\Delta\rho_{\beta,2}$[**E-Br** IV]
$\nu_{\beta,2} = \pm 0.510$
$\Delta E_{\beta,2} = -174$

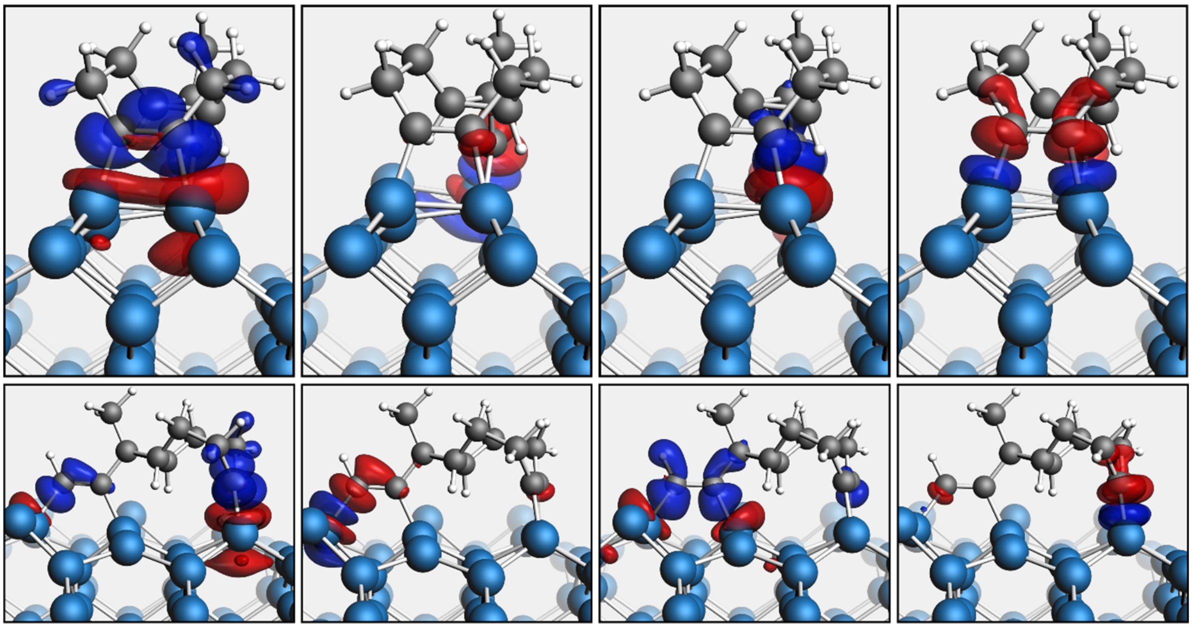

$\Delta\rho_{\alpha,3}$[**C-OT** III + **E-Br** I/III]
$\nu_{\alpha,3} = \pm 0.642$
$\Delta E_{\alpha,3} = -324$

$\Delta\rho_{\beta,3}$[**E-Br** II]
$\nu_{\beta,3} = \pm 0.479$
$\Delta E_{\beta,3} = -149$

$\Delta\rho_{\alpha,4}$[**E-Br** III]
$\nu_{\alpha,4} = \pm 0.608$
$\Delta E_{\alpha,4} = -276$

$\Delta\rho_{\beta,4}$[**C-OT** IV]
$\nu_{\beta,4} = \pm 0.453$
$\Delta E_{\beta,4} = -160$

*Figure S17: NOCV deformation densities for **DB(OT+BR)** based on the electron spin (α or β). Electron accumulation shown in blue and electron depletion in red. Contributions to $\Delta E_{orb}$ (ΔE) in kJ·mol$^{-1}$.*

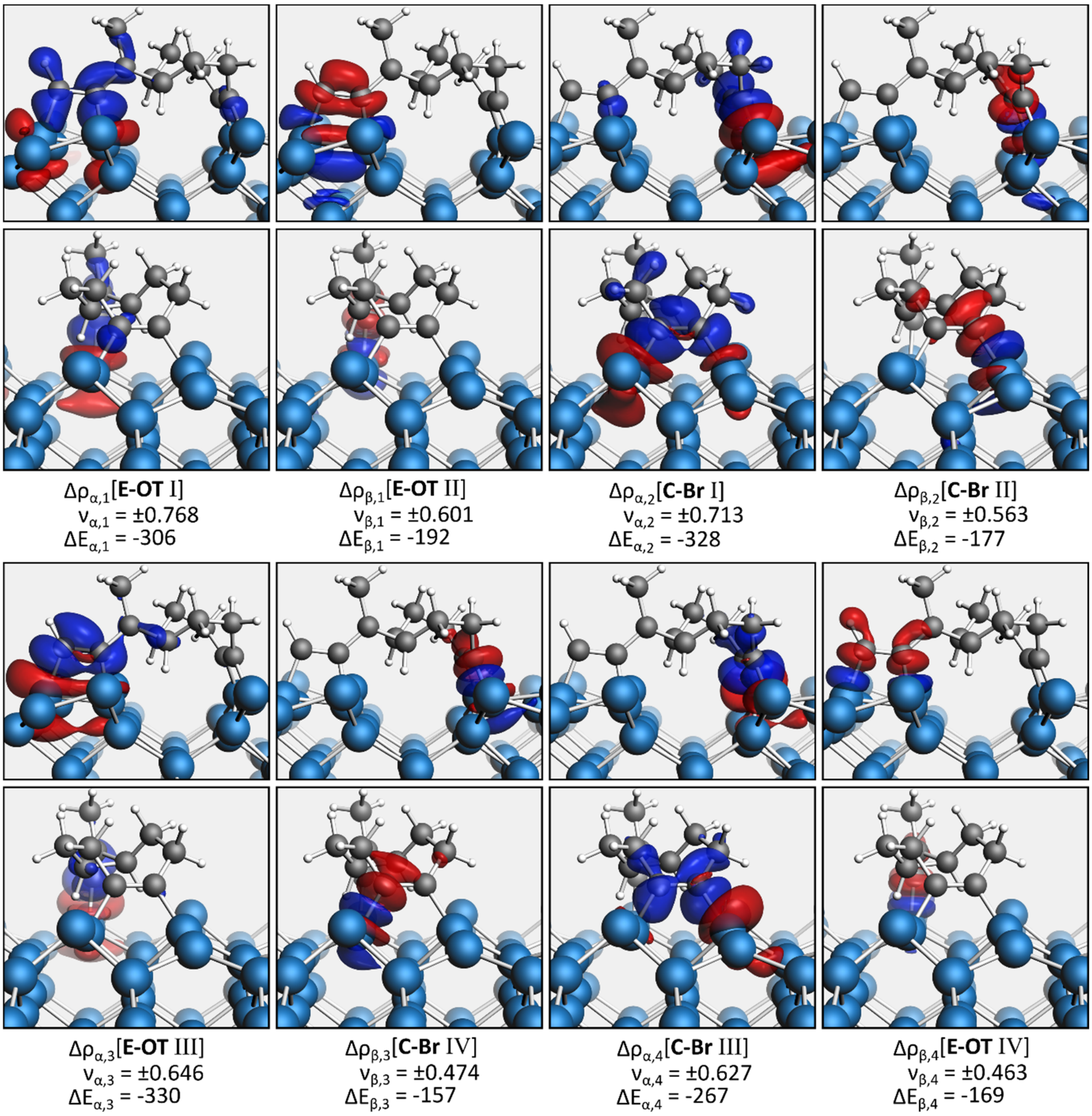


*Figure S18: NOCV deformation densities for **C-Br + E-OT** based on the electron spin (α or β). Electron accumulation shown in blue and electron depletion in red. Eigenvalues (ν) in $q_e$ and contributions to $\Delta E_{orb}$ (ΔE) in kJ·mol$^{-1}$.*

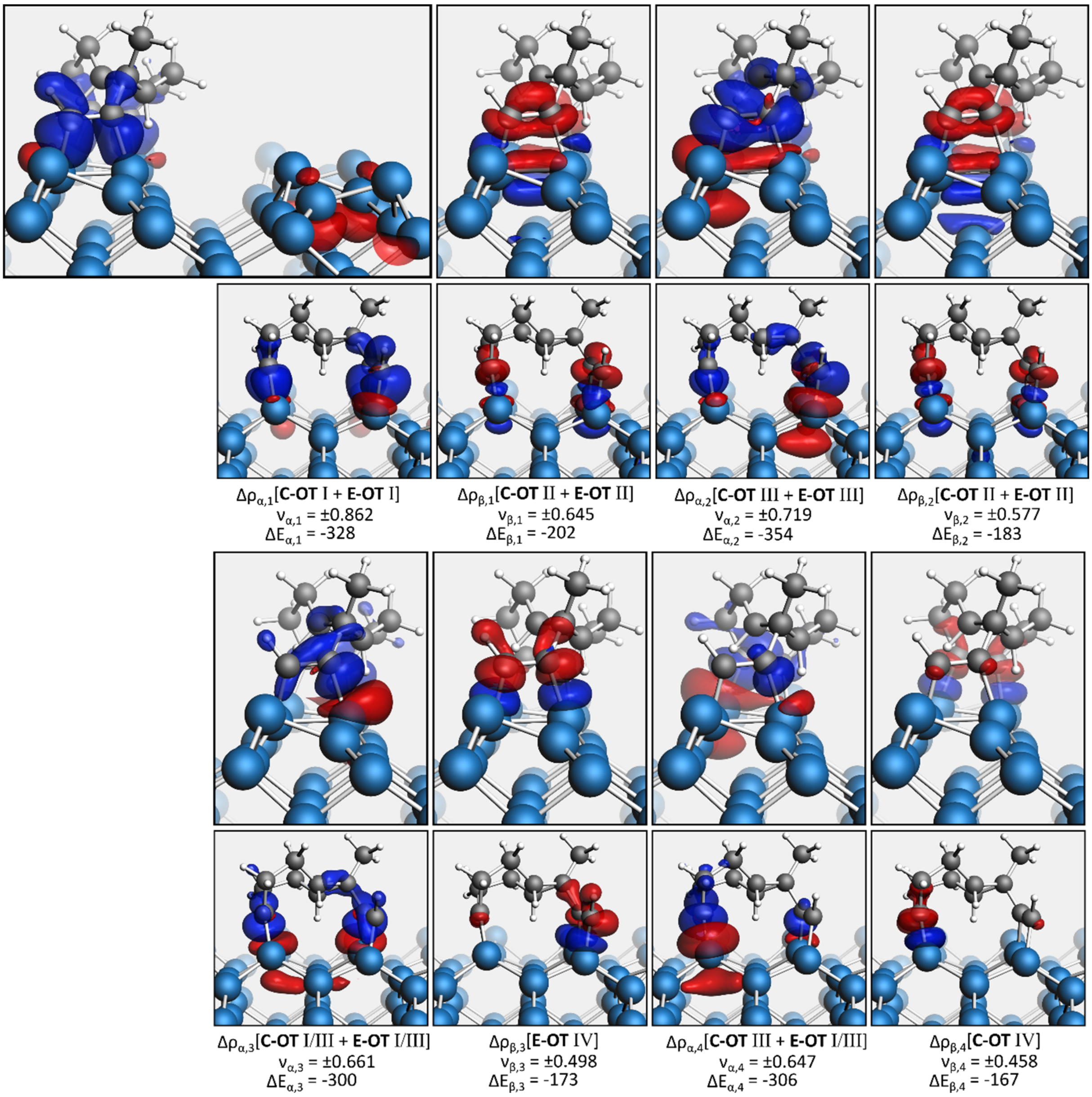


*Figure S19: NOCV deformation densities for **C-OT + E-OT** based on the electron spin (α or β). Electron accumulation shown in blue and electron depletion in red. Eigenvalues (ν) in $q_e$ and contributions to $\Delta E_{orb}$ (ΔE) in kJ·mol$^{-1}$. For the first deformation density an unreasonable electron transfer is observed from the neighboring dimer row. This is a result from nearly degenerate states for the surface fragment. Although this one deformation density is thereby biased, the overall picture is reliable.*

## Ab-initio Molecular Dynamic simulations

The probability to observe a reaction event in an AIMD calculation can be estimated from the Arrhenius equation:

$$k = A \cdot e^{-\frac{E_A}{R \cdot T}}$$

For the following estimation we use a temperature (T) of 300 K and a preexponential factor (A) of $6{\cdot}10^{12}$ $s^{-1}$ as found for cyclooctyne.[1] Based on the reaction barrier ($E_A$) it is now possible to roughly estimate how likely the observation of this reaction event is. However, the estimate in **Table S4** assumes the absence of any competing reaction path. In case several reaction events are possible one would need to average the probability for one certain event based on its contribution to the sum of the reaction constants.

*Table S4: Estimation of the probability to observe a reaction event in an AIMD simulation based on the reaction barrier. Reaction constants are estimated at 300 K and with a preexponential factor of $6{\cdot}10^{12}$ $s^{-1}$.*

| Reaction barrier $E_A$ [kJ·mol$^{-1}$] | Reaction constant k [s$^{-1}$] | Reaction events in 15ps | Sampling time for 1 event observation |
|---|---|---|---|
| 10 | 108900677017 | 1634 | 0.009 ps |
| 20 | 1976559576 | 30 | 0.5 ps |
| 30 | 35874779 | 0.54 | 28 ps |
| 37 | 2167728 | 0.03 | 461 ps |
| 40 | 651131 | 0.01 | 1536 ps |


[1] L. Pecher, S. Schmidt, R. Tonner, *J. Phys. Chem. C* **2017**, *121*, 26840–26850.

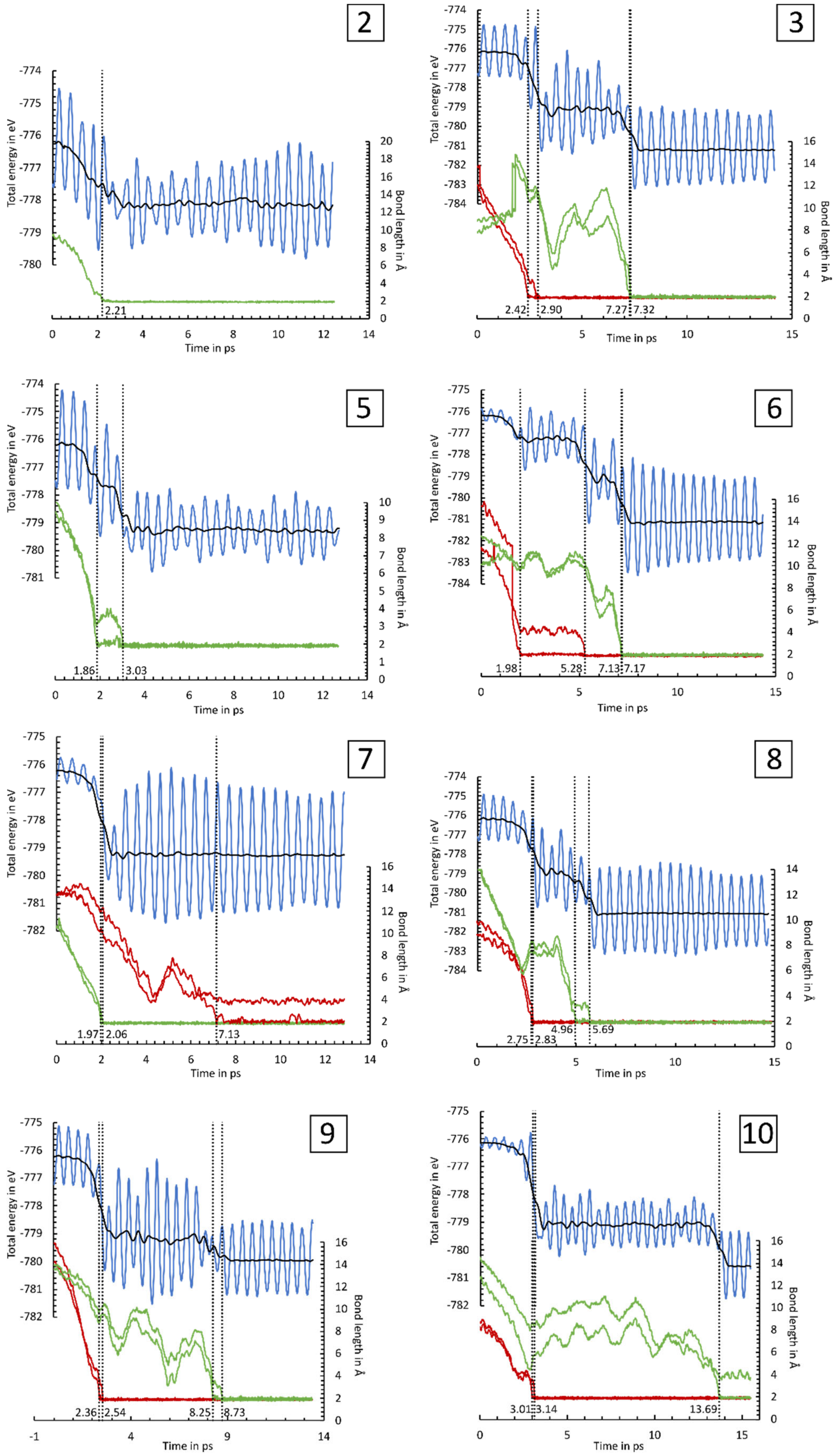


*Figure S20: Energy (blue line) and C-Si bond length (green line: cyclooctyne group; red line ethinyl group) changes for the MD simulation 2-3 and 5-10. The two-sided moving average of the energy in shown as black line. Dotted lines indicate the C-Si bond formation, stated with the time frame.*
